# A comparison study of human examples vs. non-human examples in an evolution lesson leads to differential impacts on student learning experiences in an introductory biology course

**DOI:** 10.1186/s12052-021-00148-w

**Published:** 2021-06-26

**Authors:** Daniel Z. Grunspan, Ryan D. P. Dunk, M. Elizabeth Barnes, Jason R. Wiles, Sara E. Brownell

**Affiliations:** 1 Department of Integrative Biology, University of Guelph, Guelph, Canada; 2 School of Biological Sciences, University of Northern Colorado, Greeley, USA; 3 Department of Biology, Middle Tennessee State University, Murfreesboro, USA; 4 Department of Biology, Syracuse University, Syracuse, USA; 5 Research for Inclusive STEM Education Center, School of Life Sciences, Arizona State University, Tempe, USA

**Keywords:** Evolution Education, Human examples

## Abstract

**Background:**

Instructors can teach evolution using any number of species contexts. However, not all species contexts are equal, and taxa choice can alter both cognitive and affective elements of learning. This is particularly true when teaching evolution using human examples, a promising method for evolution instruction that nevertheless comes with unique challenges. In this study, we tested how an evolution lesson focused on a human example may impact students’ engagement, perceived content relevance, learning gains, and level of discomfort, when compared to the same lesson using a non-human mammal example. We use this isomorphic lesson and a pre-post study design administered in a split-section introductory biology classroom to isolate the importance of the species context.

**Results:**

For two of the four measurements of interest, the effect of using human examples could not be understood without accounting for student background. For learning gains, students with greater pre-class content knowledge benefited more from the human examples, while those with low levels of knowledge benefited from the non-human example. For perceived relevance, students who were more accepting of human evolution indicated greater content relevance from the human example. Regardless of condition, students with lower evolution acceptance reported greater levels of discomfort with the lesson.

**Conclusions:**

Our results illustrate the complexities of using human examples to teach evolution. While these examples were beneficial for many students, they resulted in worse outcomes for students that were less accepting of evolution and those who entered the course with less content knowledge. These findings demonstrate the need to consider diverse student backgrounds when establishing best practices for using human examples to teach evolution.

**Supplementary Information:**

The online version contains supplementary material available at 10.1186/s12052-021-00148-w.

## Background

Instructors can use any species to illustrate evolutionary ideas in their classroom. Some species offer canonical examples of evolution, like Darwin’s finches or peppered moths, providing historical perspectives and visible examples of evolutionary processes, while other species may be more locally relevant or may be more familiar to students and present less cognitive load when learning about them. Alternatively, the instructor may choose a species to focus on because they have a greater level of expertise about that species. Because certain examples may be more effective than others, understanding how the taxa used during evolution instruction impacts students is an important step in refining evidence-based recommendations.

A promising avenue for improving evolution instruction is to use more human examples (Hillis 2007; Pobiner 2012). Teaching evolution through human examples caters to the interests of many students. However, it also amplifies cognitive and affective obstacles to learning (Pobiner et al. 2018), especially for students who find human evolution to be particularly contentious. These complications make it difficult to determine whether, when, and how to integrate human examples into evolution instruction in an evidence-based manner.

### Human examples can benefit evolution instruction

Many students prefer learning about humans compared to other taxa. Some evidence for this comes from instructors’ own classroom experiences, indicated by student course evaluations and results from informal survey questions (Werth 2009; Wilson 2005). Using more general survey sampling methods, Paz-y-Mino and Espinosa (2009) found that 78% of students from a sample of 461 reported a preference for an evolution course including human examples over one that focuses on plants and animals. This preference may stem from the inherent personal relevance of learning about humans (Pobiner 2012; Wilson 2005) or greater familiarity compared to other taxa (Seoh et al. 2016). This relevance is broad, helping students better understand human diversity and uniqueness, human health and disease, human origins, and what it means to be human, more generally (Alles and Stevenson 2003; Boyd et al. 2000; Donovan et al. 2019; Larsen 2014; Nesse and Williams 2012; Stearns et al. 2010).

By increasing the relevance and familiarity of evolution to students, the use of human examples can help motivate the learning process (Keller 1987) while providing a way to bridge the gap between course content and students’ lived experiences. Recently, the Teaching Evolution through Human Examples (TEtHE) project developed and tested a human-focused evolution curriculum in high school level AP Biology classrooms, showing a trend of improved learning gains and evolution acceptance across the ten classrooms where it was implemented (Pobiner et al. 2018). Importantly, and discussed in more detail below, the success of this human-focused curriculum relied on the use of cultural and religious sensitivity teaching strategies. These studies, along with others (e.g. Hillis 2007; Pobiner 2012), provide reasons to be optimistic about using human examples.

Establishing that students can effectively learn from human examples is an important foundation, but classroom studies to date have not identified whether human examples provide added value compared to other taxa that could be used. To date, the best comparisons between human examples and other taxa come from research on evolutionary misconceptions, where a small but growing number of studies have found contradicting results regarding how human examples impact students’ evolutionary misconceptions. In some cases, human examples may lead to more accurate applications of natural selection. For example, students were better able to successfully subvert misconceptions about natural selection rooted in essentialist biases when thinking about humans instead of other animal or plant species (Nettle 2010; Shtulman and Schulz 2008). In an experimental study with 50 undergraduate students, Nettle (2010) found that, when presented pictures of humans, animals, or inanimate objects, college students in the UK were better able to recognize variation in pictures of humans. These same students were less prone to several evolutionary misconceptions when questions were framed around a hypothetical human population as opposed to a hypothetical non-human population (Nettle 2010). However, human contexts have also been shown to increase the use of misconceptions compared to non-human contexts, including a greater propensity for students to attribute the evolution of new traits to their use or disuse (Ha et al. 2006). It is not entirely clear why human examples mitigate misconceptions in some circumstances but exacerbate them in others.

### Students are differentially impacted by human examples

Another important consideration is that the influence of human examples on cognitive processes differs between students. For example, the saliency of the species context used is greater for novices compared to experts. When asked to sort problems based on different features, novices in evolutionary biology frequently sorted based on species context, including whether the problem included humans (Nehm and Ridgway 2011). This occurred even though the species context did not change the basic problem structure. Experts performing the same task never sorted based on species context, suggesting that the effect of species context on the way one approaches evolutionary problems may dampen with greater content knowledge and improved abilities to think conceptually about the problem. Smith et al. (2013) similarly found that non-majors biology students frequently sorted cards based on taxonomic differences while biology faculty did not. The decreased salience of species on evolutionary reasoning as expertise is gained has also been found when comparing answers to evolutionary problems between children and adults, where children’s responses are more prone to the taxa included in the question (Shtulman and Schulz 2008). Thus, students with lower conceptual knowledge of evolution may be particularly prone to any advantages or disadvantages that stem from using a human context.

Differential experiences may also exist based on student evolution acceptance. Evolution rejection is notoriously problematic in the US (Miller et al. 2006) and certain religious beliefs underlie many student conflicts (Barnes et al. 2019; Dunk et al. 2017; Glaze et al. 2015). Polls highlighting evolution rejection typically ask about human evolution (Brenan 2019), where rejection tends to be high (Nadelson and Southerland 2012). However, distinguishing between microevolution, macroevolution, and human evolution illustrates that most individuals accept microevolution, while human evolution is less commonly accepted (Barnes et al. 2020a; Ranney and Thanukos 2011; Scott 2008). For students who reject human evolution, but otherwise accept evolution of non-human species, human examples may lead to greater levels of discomfort, decreased engagement, and ultimately reduced learning compared to using other taxa.

To date, only a few empirical studies have considered the ideological and educational backgrounds of participants when testing the impact of human examples on evolution instruction. The development of curriculum as part of the previously mentioned TEtHE project included resources on cultural and religious sensitivity (Pobiner et al. 2018). These practices aimed to manage conflict between students’ cultural and religious backgrounds and science; use of these resources was imperative to the efficacy of instruction (Bertka et al. 2019; Pobiner et al. 2018). This finding mirrors other work highlighting the importance of religious cultural competence in evolution education (Barnes et al. 2020b; Barnes and Brownell 2017). Another recent study examined student reasoning and knowledge between anthropology and biology majors, including how well students could identify key concepts, how often they expressed naïve ideas, their use of scientific models, and their knowledge of natural selection (Beggrow and Sbeglia 2019). Biology and anthropology students were demographically distinct from one another and were differentially impacted by the use of human versus non-human examples. While switching taxa did not affect biology students, anthropology students expressed fewer key concepts and were less likely to answer questions using a pure scientific model when addressing human examples (Beggrow and Sbeglia 2019).

### The current study

While there are reasons to be excited about using human examples teaching evolution, a more thorough understanding of their impacts on student experiences will help refine evidence-based guidelines for their use. This includes understanding their value compared to other taxa and how student background may moderate learning experiences.

We advance research on human examples in evolution instruction in two ways. First, we test the value added by using human examples compared to non-human mammalian examples. Second, we test whether any impacts of the species context are dependent on student backgrounds. We use a controlled quasi-experiment implemented in two iterations of the same split-section large introductory biology classroom. We isolate the effect of species taxa by using different versions of the same single-day active learning lesson in each of the lecture sections, one version that includes humans and one that includes non-human mammalian taxa. Using a pre-post survey design, we examine whether the species context used during instruction affects student learning gains, perceived relevance of the lessons’ content, engagement with the lessons’ content, and discomfort with the lessons’ content. Further, we test for moderating effects between the taxa used during the lesson and students’ prior evolution knowledge and acceptance of human evolution. We predicted that:

1. Human examples will be associated with greater learning gains than non-human animal examples.
  - This association will be moderated by prior knowledge of class content and human evolution acceptance; students with more prior knowledge of class content and more accepting of human evolution will experience greater learning gains in the human example.
2. Human examples will be associated with greater perceived relevance of the lesson content than non-human animal examples.
  - This association will be moderated by human evolution acceptance; students more accepting of human evolution will perceive a greater relevance from the human example.
3. Human examples will be associated with greater student engagement with the lesson content than non-human animal examples.
  - This association will be moderated by human evolution acceptance; students more accepting of human evolution will be more engaged during the lesson taught using human examples.
4. Human examples will be associated with greater student discomfort with the lesson content than non-human examples for students with lower acceptance levels of human evolution.

## Methods

### Course description

The study took place over two consecutive years in the fall semester of the same introductory biology course at a large private university in the Northeast. In both years, the course was split into morning and afternoon lecture sections. Each section was taught by the same instructor. In Year 1, 620 students were enrolled in the class, and 410 students were enrolled in Year 2. The course is required for students majoring in biology and related fields, but, as the university does not offer a survey of biology course for non-majors, many students who do not intend to major in the life sciences also enroll. The course employs the widely used Campbell Biology textbook (Reece et al. 2014) and, from its syllabus description, is “the first of a two-course sequence comprising a survey of essential biological concepts ranging from the molecular level to global ecology.” According to the course description, students “explore the nature of science and the diversity of organisms within a framework of major themes including the flow and regulation of energy and information within living systems, and the central and unifying concept of evolution.”

Culturally sensitive and culturally competent practices were implemented early in the course in an effort to mitigate student perceptions of conflict between students’ religious beliefs and acceptance of evolution (Barnes and Brownell 2017; Barnes et al. 2017a; Bertka et al. 2019). Such practices included instruction in the Nature of Science, including the limitations of science to evidence and claims rooted in the natural, physical, testable world, and, perhaps more importantly, explicit examples of individuals across a diversity of religious traditions who had reconciled evolutionary science with their sincerely-held religious faith. Examples of the former include that students were presented with and given the opportunity to discuss the statements collected by the Clergy Letter Project (Zimmerman 2010), comprising clearly-worded affirmations from Christian clergy of many denominations as well as Jewish, Buddhist, Unitarian Universalist, and Humanist clergy, of the veracity and importance of evolution along with unambiguous assertions that acceptance of evolution is entirely compatible with the tenets of their religious traditions. Additionally, students were presented with specific examples of evolutionary scientists who are open and practicing Catholic, Protestant, and Evangelical Christians, other scientists who are Muslims, and religious leaders of the Hindu, Buddhist, and Christian traditions who have advocated for the compatibility of evolution and their respective faiths. Students were also presented with the Inter-Academy Panel Statement on the Teaching of Evolution (Inter-Academy Panel 2007) in which the academies of science from countries around the world expressed agreement on “evidence-based facts” which “scientific evidence has never contradicted,” including that the universe and Earth are billions of years old, that the evolution of life on Earth is also measured in billions of years, and that “all organisms living today, including humans, clearly indicate their common primordial origin.”

Evolution is used as a recurring and organizing theme throughout the course, and it is identified as such from the first day of class. Prior to the lesson at the heart of this study, students had explored evolution in the context of the origin of biological molecules, as an extension of cell theory, and as an explanation for the diversity of life. They had been assigned to read and had class discussion around the Understanding Evolution website’s sections on the patterns of evolution (University of California Museum of Paleontology 2021).

#### The lesson

To test the impacts of species context, two versions of the same phylogeny lesson were adapted for use in this study; a treatment version (human) and a control version (non-human mammals). The lesson took place over the course of a single 55-min class day. The structure of the lesson was adapted from a previously designed active learning lesson (Nelson and Nickels 2001, Additional file 1), and was typical of a normal class day. In each year, a coin flip was used to determine which section received the human version and which received the non-human mammal version. The same instructor (RDPD) taught both sections in each year to minimize any effect of instructor on student outcomes.

After a brief introduction from the instructor, students were instructed to form groups with their neighbors and work together to construct phylogenetic trees from molecular sequence data. Students were then given species names for each molecular sequence and used the phylogenies to answer questions about the different species. In the treatment version, several of the questions required students to consider humans’ placement in the phylogeny. These questions were the same in the control version, but asked about the corresponding non-human species, instead. The species in the human version of the lesson included humans, chimpanzees, gorillas, and other non-human primates. The non-human mammal version included dogs, and different species of weasel, badger, and mongoose. We chose these non-human mammals because they were likely to be familiar without evoking strong feelings, which may have been the case if the phylogeny was mostly domestic pets, charismatic megafauna, or species that students may be averse to. All materials of the lesson, including the worksheets and instructions provided to students, were identical between the treatment and control lessons other than the species names in the worksheet.

The lesson was situated in the context of students learning about phylogenetic trees in preparation for applying tree-thinking as the primary organizational scheme for exploring diversity within, and relationships among, major biological taxa. Students had some prior exposure to phylogenetic trees, including the type used in the study lesson, but they had not yet been presented with trees depicting humans in relation to other animals.

### Measurements: independent variables

Students completed a pre-class survey online that was due before class began on the morning of the lesson. Students were given several days to complete this survey. This pre-class survey included instruments to measure phylogeny content knowledge, and several measures about students’ affect toward the course in general. These included students’ level of engagement with course content, perceived relevance of the course content, and discomfort with course content. Students’ phylogeny content knowledge before the lesson was measured using ten items from the Tree Thinking Concept Inventory (TTCI) (Gibson and Hoefnagels 2015) that were most pertinent to the lessons’ content. Previously developed instruments were modified and used to measure student engagement with course content (Richmond 1990), perceived relevance of course content (Frymier and Shulman 1995), and discomfort with course content (Barnes et al. 2020a). The prompt to each of these instruments on this pre-class survey instructed students to answer items in regard to the course (Additional file 1). As a shorthand, we refer to these course-level measures as Engagement (course), Relevance (course), and Discomfort (course). This study took place after students had been in the course for several weeks, so students had time to develop opinions about the course.

Student acceptance of human evolution was measured using the Inventory of Student Evolution Acceptance (I-SEA) (Nadelson and Southerland 2012). In Year 1, students completed the entire I-SEA once during the final week of the course. In Year 2, students completed just the human evolution portion of the I-SEA as part of the pre-class survey. Because human evolution acceptance is the primary construct of interest in this study, we only analyzed the eight items making up the human evolution portion of the I-SEA. As previous research has shown, the I-SEA can function as sub-scales for acceptance of microevolution, macroevolution, and human evolution (Sbeglia and Nehm 2019).

### Measurements: dependent variables

Students were assigned an additional online survey due within three days of the phylogeny lesson. This post-class survey measured phylogeny content knowledge using the same ten items from the TTCI that students took on the pre-class survey. This survey also included the same items to measure engagement, perceived relevance, and discomfort instruments, but this time the prompts instructed students to refer to their experience with the content from the phylogeny lesson. Thus, these measures capture students’ engagement with the phylogeny lesson content, perceived relevance of the phylogeny lessons’ content, and discomfort with the phylogeny lessons’ content. As a shorthand, we refer to these as Engagement (lesson), Relevance (lesson), and Discomfort (lesson).

**Fig. 1:**
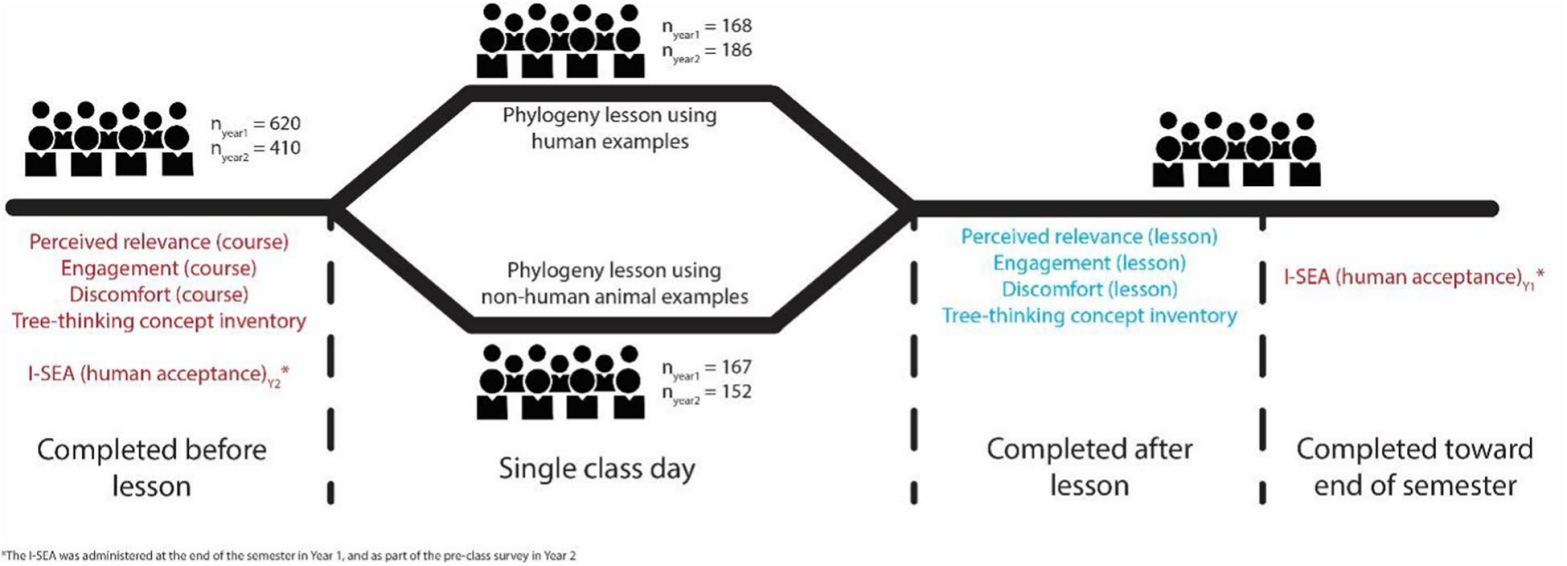
An overview of the study design, including survey instruments administered (outcome measures in blue; independent variables in red) and their timing. Differences in the timing of administration of the I-SEA between the replications of this study are shown. Sample sizes in the class day indicates the number of students that attended class the day of the lesson and completed at least one of the surveys administered

An overview of the study design can be found in Fig. 1. All research activities were approved by Syracuse University IRB, protocol #18-248.

#### Analyses

All instruments showed high internal reliability for measures regarding the course content measures about the lesson. For perceived relevance, Cronbach’s alpha was 0.88 for responses about the course and 0.90 for responses about the lesson. For engagement, α = 0.86 for responses about the course and 0.88 for responses about the lesson. For discomfort, α = 0.92 for responses about the course and 0.93 for responses about the lesson. For the human acceptance scale from the I-SEA, α = 0.91. Evidence of validity for all instruments was supported based on internal structure and the relationship of measurements to other variables (Additional file 1). Responses for content knowledge, engagement, perceived relevance, and discomfort were summed and treated as continuous variables for each student.

Multiple linear regression modeling was used to test our predictions. The first prediction, that human examples will be associated with greater learning gains than non-human animal examples, was tested by modeling the effect of treatment (human or non-human animal lesson) on post-class TTCI scores. We included pre-class TTCI scores, human evolution acceptance scores (from the I-SEA), and year (Year 1 or Year 2) as control variables in this model. The effect of treatment in this model provides an explanation of whether species context resulted in significantly different gains to TTCI scores, holding TTCI scores prior to the lesson, human evolution acceptance, and experimental year constant. Two additional models were run to test the associated predictions that the effect of species context on learning gains will be moderated by (1) students’ prior content knowledge and (2) students’ acceptance of human evolution. These models were the same as the main effects model described above but included interactions between (1) treatment and pre-class TTCI scores and (2) treatment and human evolution acceptance scores. The interaction in each of these models was used to determine whether any effect of species context on learning gains was moderated by either prior content knowledge or human evolution acceptance.

The second prediction, that human examples will be associated with greater perceived relevance of the lesson content than non-human animal examples, was tested by modeling the effect of treatment on students’ relevance (lesson) scores. In this model, we included relevance (course) scores, human evolution acceptance scores, and year in the model as control variables. The effect of treatment in this model was used to test whether there was a main effect of treatment on perceived relevance, holding students’ perceived relevance of course content, human evolution acceptance, and experimental year constant. One additional model was run to test the associated prediction that the effect of species context on perceived relevance of lesson content will be moderated by students’ acceptance of human evolution. This model was the same as the main effects model but included an interaction between treatment and students’ acceptance of human evolution. The third prediction, that human examples will be associated with greater engagement with the lesson content than non-human animal examples, and the associated prediction that this effect will be moderated by students’ acceptance of human evolution, was tested using the same two-step modeling framework described for perceived relevance, except measures of perceived relevance of course and lesson content were replaced with measures of engagement with course and lesson content, respectively.

The fourth prediction, that human examples will be associated with greater student discomfort with the lesson content than non-human animal examples for students with lower acceptance of human evolution, was tested using logistic regression. This approach was taken based on the distribution of discomfort (course) and discomfort (lesson) scores. Discomfort scores of zero and four were extremely common, resulting in a bimodal distribution not suitable for typical linear regression (Additional file 1: Figure S1). Discomfort (lesson) scores were dichotomized so students with a score greater than zero were coded as 1 and students with a score of zero as 0. Using these dichotomized scores, logistic regression was used to model the probability that a student reported any level of discomfort with the lesson content above zero, with treatment, discomfort (course), human evolution acceptance scores, year, and an interaction between treatment and human evolution acceptance scores modeled as predictors variables. The effect of this interaction was used to test whether there was a moderating effect of human evolution acceptance on the association between species context and discomfort with the lesson content. An additional main effects model without the interaction was run to help interpret the interaction coefficient.

### Missing data and multiple imputation

Complete responses to all items in the pre- and post-surveys were uncommon. After accounting for issues with non-response, 145 out of 620 consenting students from Year 1 and 128 out of 410 consenting students from Year 2 provided complete case data (40.8% of the consenting students).

To handle issues with missing data, the regression analyses described above were run two different ways. First by using listwise deletion and then using multiple imputation methods. For the imputation, we assumed that data were missing at random (MAR) (Rubin 2004), because class grades, which were available for nearly every student, were negatively associated with data missingness. A Mann–Whitney test indicated that students with complete data for the surveys performed significantly better in the course than those with missing data, U = 69,678, p < 0.001. Multiple imputation was run with a fully conditional specification (van Buuren 2007, 2018) in *R* using the *mice* package (van Buuren et al. 2011). Predictive mean matching (pmm) was used to calculate imputed values of all variables (Andridge and Little 2010). All variables were imputed at the level of the sum score. Because interactions were of theoretical interest, imputations were performed separately for students in the human arm and students in the animal arm of the study. One hundred datasets were imputed for each subset before being recombined into the final imputed datasets. Diagnostics for convergence and model fit were performed before analyses were performed on the imputed data (Additional file 1: Figures S3–S14). Regression model results were pooled according to recommended guidelines (Rubin 2004).

Model estimates were similar between listwise deletion and the imputed datasets for all models. However, because of uncertainty involved in analyses with missing data, we report regression results for both methods.

## Results

### Fit of imputed values

The fit of the imputation model was evaluated by examining differences between observed and imputed data. In general, the mean values for the Tree Thinking Concept Inventory (TTCI), perceived relevance, engagement, discomfort, and human evolution acceptance were similar between imputed and complete cases values (Table 1). However, there were some exceptions. The mean imputed value for missing TTCI scores on the post-survey was higher than the mean complete cases TTCI score in the animal arm of the experiment but was lower than complete cases in the human arm. Students were less likely to complete the post-survey TTCI in the human arm compared to the animal arm. Imputed values for the discomfort survey were higher than complete cases for both pre- and post-surveys in both arms of the experiment. Lastly, the mean imputed human acceptance score was higher than the mean score for complete cases in the human arm, while the means in the animal arm were identical. For all measures, student data were more likely to be missing from measures on the post-survey compared to measures on the pre-survey. Diagnostic checks of imputed values suggest reasonable estimates for all measures of interest (Additional file 1: Figures S3–S14).

**Table 1:** Comparison of summary data from the listwise deletion and imputed data

		Content Knowledge		Relevance		Engagement		Discomfort		I-SEA: Human Evo	Class Grade
		(0–11)		(0–24)		(0–20)		(0–12)		(0–32)	(0–4.0)
		Pre	Post	Course	Lesson	Course	Lesson	Course	Lesson	-	-
Animal	Listwise Deletion	3.93 (1.94) (n = 249)	4.06 (1.93) (n = 186)	13.4 (4.71) (n = 252)	14.3 (4.40) (n = 187)	11.5 (4.52) (n = 248)	11.2 (4.16) (n = 188)	1.96 (2.37) (n = 252)	2.28 (2.64) (n = 187)	22.8 (5.98) (n = 263)	3.04 (0.91) (n = 319)
	Imputed Data	3.70 (n = 70)	4.19 (n = 133)	13.3 (n = 67)	14.7 (n = 132)	11.4 (n = 71)	11.6 (n = 131)	2.46 (n = 67)	2.58 (n = 132)	22.8 (n = 56)	No missing values
Human	Listwise Deletion	3.94 (2.03) (n = 269)	4.59 (2.39) (n = 173)	13.3 (4.25) (n = 271)	13.9 (4.62) (n = 172)	11.5 (4.38) (n = 269)	10.9 (4.61) (n = 171)	1.87 (2.21) (n = 270)	1.76 (2.31) (n = 172)	23.9 (5.69) (n = 284)	3.15 (0.79) (n = 354)
	Imputed Data	3.94 (n = 85)	4.30 (n = 181)	13.3 (n = 83)	13.9 (n = 182)	11.2 (n = 85)	10.9 (n = 183)	1.99 (n = 84)	2.09 (n = 182)	26.9 (n = 70)	No missing values

Means and number of data points are included for both listwise deletion and imputed data. Standard errors for the listwise deletion data are in parentheses

### Learning gains

Treatment was not a significant predictor of post-class TTCI scores in the main effects model for both listwise deletion and imputation analyses (Additional file 1: Table S2), indicating no relationship between species context on the phylogeny lesson and post-class scores keeping pre-class TTCI score, human evolution acceptance, and experimental year constant. The only significant predictor of post-class TTCI scores in the main effects model was pre-class TTCI score.

However, a significant interaction was found between treatment and pre-class TTCI score for both listwise deletion (*β* = 0.28, *t*(276) = 2.764, *p* = 0.006) and multiple imputation (*β* = 0.25, *t*(219.8) = 2.688, *p* = 0.008) (Table 2). This interaction suggests that the species context moderated the effect of pre-class knowledge on learning gains. The human lesson was disproportionately associated with greater learning gains for students with higher pre-class TTCI scores compared to students with lower pre-class TTCI scores.

**Table 2:** Multiple linear regression model results for post-class tree thinking concept inventory scores

	TTCI Post-Score (Model 1)				TTCI Post-Score (Model 2)			
	*CC*		*MI*		*CC*		*MI*	
	*Β* (Std. error)	p-value	*Β* (Std. error)	p-value	*Β* (Std. error)	p-value	*Β* (Std. error)	p-value
Intercept	**1.96** **(1.44 – 2.49)**	** < 0.001**	**1.895** **(1.66 – 2.13)**	** < 0.001**	**2.50** **(1.86 – 3.14)**	** < 0.001**	**2.428** **(2.14–2.72)**	** < 0.001**
TTCI Pre-Score	**0.59** **(0.49 – 0.69)**	** < 0.001**	**0.606** **(0.558 – 0.654)**	** < 0.001**	**0.46** **(0.32 – 0.60)**	** < 0.001**	**0.468** **(0.40 - 0.54)**	** < 0.001**
Year (Reference: Year 1)	− 0.40 (− 0.82 to 0.01)	0.059	− 0.22 (– 0.39 to – 0.05)	0.197	− **0.41** **(**– **0.83 to 0.00)**	**0.050**	− 0.254 (− 0.42 – 0.09)	0.134
I-SEA_H_ (Centered)	0.02 (− 0.03 to 0.08)	0.393	0.032 (0.01 – 0.06)	0.196	0.01 (– 0.03 to 0.05)	0.560	0.012 (− 0.01 – 0.03)	0.502
Treatment (Reference: Animal)	0.35 (− 0.07 to 0.77)	0.102	0.281 (0.09 – 0.48)	0.153	− 0.77 (– 1.65 to 0.12)	0.090	− 0.692 (– 1.094 to − 0 .29)	0.086
Treatment X I-SEA_H_	− 0.03 (− 0.11 to 0.05)	0.477	− 0.042 (− 0.08 − 0.01)	0.231	-	-	-	-
Treatment X TTCI Pre-Score	-	-	-	-	**0.28** **(0.08 – 0.47)**	**0.006**	**0.249** **(0.157 – 0.342)**	**0.008**
Observations	282		673		275		673	
R^2^ / adjusted R^2^	0.343/0.331				0.376 / 0.364			

Full model results predicting student TTCI post-scores using complete-case data (CC) and multiple imputation (MI). Year and treatment are binary variables with reference values in parentheses. MI results are pooled following Rubin (2004). Coefficients significant at p ≤ 0.05 are bolded

The interaction between treatment and human evolution acceptance was not significant using either listwise deletion or multiple imputation.

### Engagement

Treatment was not a significant predictor of student engagement with lesson content (Additional file 1: Table S3). The interaction between treatment and human evolution acceptance was also not significant (Table 3). Instead, the only variable that significantly predicted engagement with the lesson content was engagement with the course content. Model results for listwise deletion and the imputed datasets were comparable.

**Table 3:** Multiple linear regression model results for reported engagement and relevance scores during class activity

	Engagement with lesson content				Perceived relevance of lesson content			
	*CC*		*MI*		*CC*		*MI*	
	*Β* (Std. error)	p-value	*Β* (Std. error)	p-value	*Β* (Std. error)	p-value	*Β* (Std. error)	p-value
Intercept	**4.86** **(3.49 – 6.23)**	** < 0.001**	**5.024** **(4.332 – 5.72)**	** < 0.001**	**8.04** **(6.43 – 9.66)**	** < 0.001**	8.404 (7.64 – 9.17)	** < 0.001**
Engagement with course content	**0.58** **(0.49 – 0.68)**	** < 0.001**	**0.567** **(0.52 – 0.62)**	** < 0.001**	**-**	**-**	**-**	**-**
Perceived relevance of course content	**-**	**-**	**-**	**-**	**0.49** **(0.38 – 0.59)**	** < 0.001**	**0.45** **(0.40 – 0.50)**	** < 0.001**
Year (Reference: Year 1)	− 0.51 (– 1.36 to 0.35)	0.248	− 0.319 (-0.71 – 0.07)	0.409	− 0.46 (– 1.40 to 0.47)	0.333	− 0.141 (– 0.53 – 0.25)	0.716
I-SEA (Centered)	− 0.05 (– 0.15 to 0.06)	0.359	0.01 (– 0.04 – 0.06)	0.838	− 0.09 (– 0.20 to 0.03)	0.157	− 0.089 (– 0.14 – 0.04)	0.083
Treatment (Reference: Animal)	− 0.37 (– 1.20 to 0.47)	0.389	− 0.431 (– 0.83 – 0.03)	0.283	− 0.29 (–1.23 to 0.65)	0.544	− 0.455 (– 0.89 – 0.02)	0.299
Treatment X I-SEA	0.06 (– 0.10 to 0.22)	0.474	− 0.021 (– 0.09 – 0.05)	0.765	**0.18** **(0.00 – 0.36)**	**0.048**	0.148 (0.07 – 0.22)	0.052
Observations	281		673		285		673	
R^2^ / adjusted R^2^	0.366/0.355				0.247/0.234			

Full model results predicting student engagement and relevance post-scores using complete-case data (CC) and multiple imputation (MI). Year and treatment are binary variables with reference values in parentheses. MI results are pooled following Rubin (2004). Coefficients significant at p ≤ 0.05 are bolded

### Relevance

Treatment was not a significant predictor of perceived relevance of the lessons’ content (Additional file 1: Table S3), holding all other variables constant. However, a significant interaction between treatment and human acceptance levels was found in the full model using listwise deletion (*β* = 0.18, *t*(279) = 1.99, *p* = 0.048) (Table 3). The effect size for this interaction using multiple imputation data was comparable, but not significant at α = 0.05 (*β* = 0.15, *t*(191.3) = − 1.06, *p* = 0.052) (Table 3). This interaction suggests that the association between species context and perceived relevance during the lesson was moderated by the level to which a student accepts human evolution, where students with lower levels of human evolution acceptance perceived greater relevance from the animal lesson while students with higher levels of acceptance perceived greater relevance from the human lesson.

### Discomfort

Out of 359 students who completed the post-class discomfort items, 175 reported a score of zero, while the other 184 students had an average discomfort score of 4.17, with most of the non-zero responses having a score of 4. Given this distribution, we used logistic regression to test the likelihood of students reporting discomfort scores greater than zero.

There was no significant effect of treatment on the likelihood a student reported a discomfort score greater than zero during the lesson, nor was there evidence of a moderating effect of human evolution acceptance on the relationship between treatment and reporting a score of discomfort greater than zero during the lesson (Table 4, Additional file 1: Table S4). However, model results indicate that students with lower levels of human evolution acceptance were more likely to report discomfort above zero during the lesson, controlling for discomfort (course), year, and treatment. This significant association was found using both listwise deletion and multiple imputation.

**Table 4:** Logistic regression model results for discomfort experienced during the one-day activity

	Discomfort with lesson content			
	Complete case data		Multiple imputation	
	Est (std)	p-value	Est (std)	p-value
Intercept	**0.16** **(0.09–0.28)**	** < 0.001**	**0.171** **(– 1.13 – 1.47)**	** < 0.001**
Discomfort with course content	**1.88** **(1.61–2.23)**	** < 0.001**	**1.9** **(0.82 – 2.98)**	** < 0.001**
*Year* 2 (Reference: Year 1)	1.32 (0.71–2.46)	0.380	1.269 (– 0.01 – 2.55)	0.338
*I-SEA* (Centered)	**0.92** **(0.87–0.99)**	**0***.***018**	**0.939** **(– 0.09 – 1.97)**	**0.019**
Treatment – Human (Reference: Animal)	0.88 (0.47–1.62)	0.679	0.84 (– 0.47 – 2.15)	0.522
Observations	285		673	
R^2^ / adjusted R^2^	0.370			

Model results predicting student discomfort using complete-case data (CC) and multiple imputation (MI). Coefficients represent odds ratios. Year and treatment are binary variables with reference values in parentheses. MI results are pooled following Rubin (2004). Coefficients significant at p ≤ 0.05 are bolded. Full model results including interaction terms are available in the Additional file 1

## Discussion

We tested the effects of using human examples compared to non-human mammal examples on student learning, engagement, perceived relevance, and discomfort with the content. We experimentally isolated the effect of species context through a lesson design where the only difference between treatment and control groups was the species names included in the lesson. We further isolated the effect of species context by statistically controlling for students’ prior knowledge of the lesson content, as well as typical levels of engagement, relevance, and discomfort with the course’s content. We did not find any main effects of species context for any of these measures, but did find that human examples differentially impacted students’ (1) learning gains based on their prior knowledge and (2) perceived relevance based on the level to which they accept human evolution. We also found that students less accepting of human evolution were more likely to report some level of discomfort with the lesson’s content, regardless of species context.

Species context was differentially associated with learning gains based on students’ prior knowledge. While we are unable to elucidate specific reasons for this pattern from our data, one possible explanation is that these students with low pre-class TTCI scores did not fully comprehend threshold concepts. A threshold concept is one that transforms a learner’s perspective in a way that enables them to see the world in a new light; this includes the ways in which they understand and interpret disciplinary content (Meyer and Land 2003). For example, threshold concepts that have been identified for understanding natural selection include randomness, probability, and temporal and spatial scales (Fiedler et al. 2017; Ross et al. 2010; Tibell and Harms 2017); fully grasping these concepts can transform one’s understanding of natural selection and lead to a changed worldview.

To date, threshold concepts for tree thinking have not been identified. However, concepts previously identified as key for tree thinking represent possible candidates; for example, understanding that there is no “ladder of progress” in a phylogenetic tree (Omland et al. 2008). It is possible that fully appreciating what can be learned from human examples of evolution is not possible without understanding these concepts. This could explain the finding that prior knowledge moderates the effect of species context on learning if students who grasped certain concepts before the start of the lesson were better prepared to integrate content from the human lesson into their understanding, and also scored higher on the pre-class TTCI. Prior research supports a connection between species context and threshold concepts and natural selection. Students referenced threshold concepts at different rates depending on whether the question was about bacteria, salamanders, or cheetahs (Göransson et al. 2020). Further research would be needed to test whether prerequisite comprehension of specific concepts promotes learning gains from human examples.

### Implications for research and teaching

This study demonstrates that human examples may improve the experiences of some students while simultaneously damaging the experiences of others. Using human examples in an evidence-based manner may require mitigating these negative experiences. To this end, several promising avenues exist. For example, teaching the nature of science (Scharmann 2018) and implementing culturally sensitive and culturally competent teaching practices have been shown to reduce perceived conflicts between students’ religious beliefs and evolution (Barnes et al. 2017b; Barnes and Brownell 2018; Barnes and Brownell 2017; Bertka et al. 2019; Pobiner 2016). Integrating these practices into evolution classrooms can be key when integrating human examples into evolution instruction (Bertka et al. 2019). More specifically, by breaking down barriers in the relationship between a students’ belief system, identity, and classroom content, these practices may be an important precondition for many students, but particularly religious students, to realize the potential benefits of human examples.

Students’ acceptance of evolution did play a role in how students experienced the phylogeny lessons, a result that occurred despite the use of culturally sensitive and competent practices. However, these findings are not evidence that these practices do not work. This study was not designed to test the impact of culturally sensitive and competent practices on student outcomes. Given prior research on the importance of these practices (Bertka et al. 2019; Truong et al. 2018), we hypothesize that the effect of evolution acceptance would have been greater in their absence. This is noteworthy, as instructors at secular institutions often do not address religion and evolution acceptance in evolution classrooms (Barnes and Brownell 2016; Dunk et al. 2019).

While it is important for instructors to consider their students’ level of evolution acceptance, it is similarly important for research exploring the impacts of human examples on evolution instruction to consider participants’ evolution acceptance. Studying the effect of human examples on student outcomes is made complex by inherent variation within and between classrooms, among other properties of curricular design and classroom structure. All else being equal, the degree to which student background moderates the effect of species context on outcomes should be expected to vary between populations. This research was performed in a classroom where I-SEA scores were higher than those found in other US institutions (Barnes et al. 2019). Similar studies with student populations less accepting of human evolution may find stronger effects. Expanding this work into different classroom populations at different institutions will be an important step moving forward.

### Strengths and limitations

This study is among the most tightly controlled tests of species context on student outcomes in evolution instruction. By controlling for differences in both instruction and student background, we were able to test for causal impacts of human examples on student learning, engagement, perceived content relevance, and discomfort. We hope future studies include similar measures of experimental control as research continues to build a more complex understanding of how human examples impact evolution instruction, and how this impact may differ by student background.

However, in isolating the importance of species context, several limitations were introduced. To keep the human and animal lessons identical, the lesson did not elaborate on any unique topics or relevant findings that are often discussed when teaching through human evolution examples, like recent hominin discoveries (Bayer and Luberda 2016; Yerky and Wilczynski 2014) or applications of evolution to human health and disease (Grunspan et al. 2020, 2019; Nesse and Natterson-Horowitz 2019; Stearns et al. 2010). This underutilization of content was an artificial element of the lesson brought on by the current study design and is thus a constraint that does not normally exist in curriculum design. The lesson used in this study was an adaptation of an activity that was published two decades ago (Nelson and Nickels 2001). Thus, this lesson misses certain best practices for teaching tree thinking that have been advanced in this time. While we did modify the lesson, changes were primarily made in the interest of the study design, and further modifications would likely improve the efficacy of the lesson itself. Future study designs may find ways to maintain appropriate control conditions while introducing content that is unique to human evolution. Further, the lesson design was restricted to a single-day intervention, but longer and more cohesive narratives may be critical for improved evolution instruction (Nehm et al. 2009).

The pre-post design of the study led to missing data issues that required multiple imputation methods to reduce potential bias in the results. While results from multiple imputation models and listwise deletion largely corroborated one another, the missing data in this study warrants a level of uncertainty when interpreting the results. Further, except for discomfort with the lesson content, we treated measurements in this study as continuous. While this decision was made to follow prior treatments of these measures, treating these measures as ordinal may be more appropriate. Further, modifications made to the engagement, relevance, and discomfort scales to tailor them for the current context introduced some concerns about the validity of these measurements, though we found evidence supporting the validity of our use of the modified measures. Despite these limitations, we hope results from this study motivate further studies on evidence-based uses of human examples in evolution instruction.

## Conclusion

In a single day active learning lesson about phylogeny, replacing the species context from non-human mammals to humans and primates affected student learning gains and how relevant students perceived the content to be. The direction of these effects differed depending on student backgrounds. Human examples disproportionately increased learning gains for students with more prior knowledge and were disproportionately perceived as more relevant for students more accepting of human evolution. Lower levels of human evolution acceptance were associated with more discomfort felt by students, regardless of the species context. Because these findings come from a single classroom context and analyses were performed with missing data, further research about how the use of human examples differentially impacts students with different cultural and education backgrounds is warranted. Nonetheless, it is unlikely that all students have similar experiences when learning about evolution through human examples. Instructors should consider this when deciding whether and how to include human examples into their curriculum.

## Supplementary Information

**Additional file 1**: **Figure S1**. Histograms of pre- and post- discomfort scores for each year of the study. **Figure S2**. Pearson correlations between the pre-class and post-class scores and human evolution acceptance measures using listwise-deletion data. **Figure S3**. Observed TTCI Pre- scores (blue) and imputed values for the 100 datasets (red). **Figure S4**. Observed TTCI Post- scores (blue) and imputed values for the 100 datasets (red). **Figure S5**. Observed Relevance (course)scores (blue) and imputed values for the 100 datasets (red). **Figure S6**. Observed Relevance (lesson) scores (blue) and imputed values for the 100 datasets (red). **Figure S7**. Observed Engagement (course) scores (blue) and imputed values for the 100 datasets (red). **Figure S8**. Observed engagement (lesson) scores (blue) and imputed values for the 100 datasets (red). **Figure S9**. Observed Discomfort (course) scores (blue) and imputed values for the 100 datasets (red). **Figure S10**. Observed Discomfort (lesson) scores (blue) and imputed values for the 100 datasets (red). **Figure S11**. Distribution of imputed TTCI scores for 100 datasets (red) compared to observed scores (blue). **Figure S12**. Distribution of imputed Relevance scores for 100 datasets (red) compared to observed scores (blue). RelPre refers to perceived relevance of the course content and RelPost refers to perceived relevance of the lesson content. **Figure S13**. Distribution of imputed Engagement scores for 100 datasets (red) compared to observed scores (blue). EngPre refers to engagement with the course content and EngPost refers to Engagement with the lesson content. **Figure S14**. Distribution of imputed Discomfort scores for 100 datasets (red) compared to observed scores (blue). DiscPre refers to discomfort with the course content and DiscPost refers to discomfort with the lesson content. **Table S1**. Frequency of missing data patterns. **Table S2**. Main effects model results for student post-test TTCI scores. **Table S3**. Main effects model results for student reported engagement and content relevance during the one-day activity. **Table S4**. Full model results for student reported discomfort experienced during the one-day activity.

## Data Availability

Data used in the analyses can be received upon request to the corresponding author.
