# Supplementary material for: A comparison study of human examples vs. non-human examples in an evolution lesson leads to differential impacts on student learning experiences in an introductory biology course
Source: Evolution (N Y). 2021 Jun 26;14(1):9. doi: 10.1186/s12052-021-00148-w (PMC8550192; doi:10.1186/s12052-021-00148-w)
Supplement: Supplementary file 1 — Additional file 1: Figure S1. Histograms of pre- and post- discomfort scores for each year of the study. Figure S2. Pearson correlations between the pre-class and post-class scores and human evolution acceptance measures using listwise-deletion data. Figure S3. Observed TTCI Pre- scores (blue) and imputed values for the 100 datasets (red). Figure S4. Observed TTCI Post- scores (blue) and imputed values for the 100 datasets (red). Figure S5. Observed Relevance (course)scores (blue) and imputed values for the 100 datasets (red). Figure S6. Observed Relevance (lesson) scores (blue) and imputed values for the 100 datasets (red). Figure S7. Observed Engagement (course) scores (blue) and imputed values for the 100 datasets (red). Figure S8. Observed engagement (lesson) scores (blue) and imputed values for the 100 datasets (red). Figure S9. Observed Discomfort (course) scores (blue) and imputed values for the 100 datasets (red). Figure S10. Observed Discomfort (lesson) scores (blue) and imputed values for the 100 datasets (red). Figure S11. Distribution of imputed TTCI scores for 100 datasets (red) compared to observed scores (blue). Figure S12. Distribution of imputed Relevance scores for 100 datasets (red) compared to observed scores (blue). RelPre refers to perceived relevance of the course content and RelPost refers to perceived relevance of the lesson content. Figure S13. Distribution of imputed Engagement scores for 100 datasets (red) compared to observed scores (blue). EngPre refers to engagement with the course content and EngPost refers to Engagement with the lesson content. Figure S14. Distribution of imputed Discomfort scores for 100 datasets (red) compared to observed scores (blue). DiscPre refers to discomfort with the course content and DiscPost refers to discomfort with the lesson content. Table S1. Frequency of missing data patterns. Table S2. Main effects model results for student post-test TTCI scores. Table S3. Main effects model results for student repo [file 12052_2021_148_MOESM1_ESM.docx]

Supplementary figures and tables

**
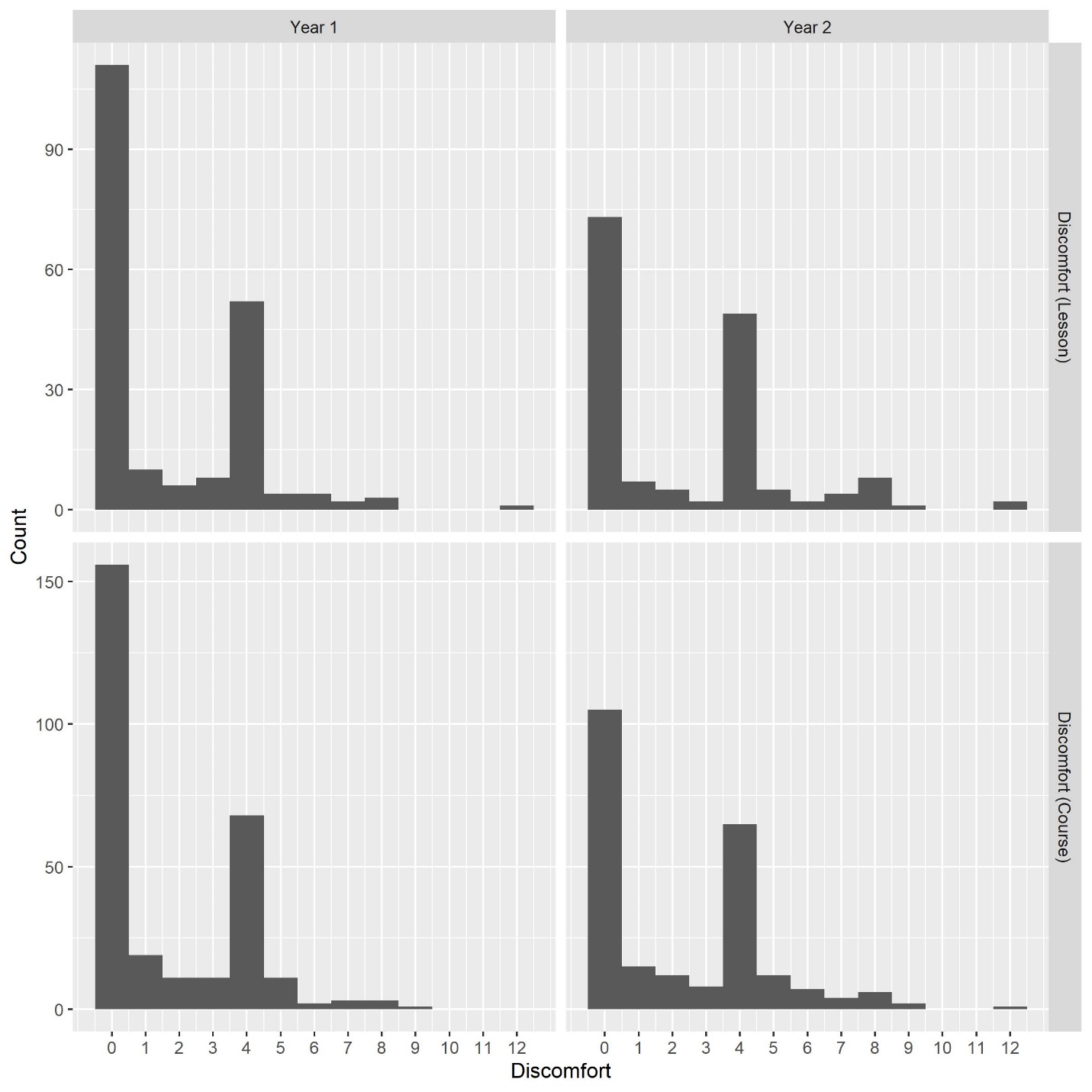
**

Supplementary Figure 1: Histograms of pre- and post- discomfort scores for each year of the study.


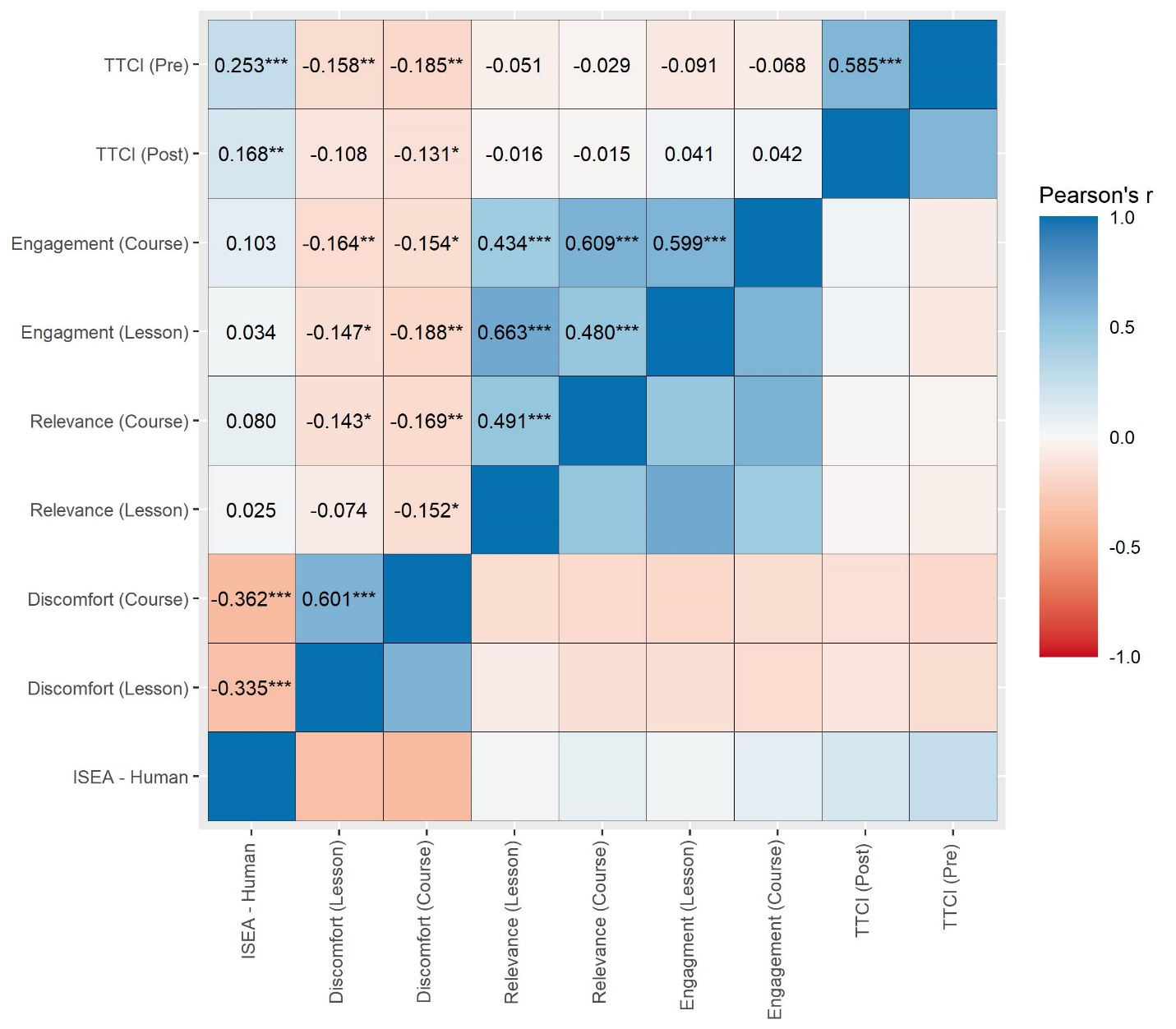


Supplementary Figure 2: Pearson correlations between the pre-class and post-class scores and human evolution acceptance measures using listwise-deletion data.

Exploratory factor analyses indicated a single factor solution for all measures, providing evidence of validity based on internal structure. For perceived relevance (course), factor loadings ranged from 0.68-0.79, with 55.5% of the variance explained by a single factor. For perceived relevance (lesson), factor loadings ranged from 0.72-0.81, with 58.6% of the variance explained by a single factor. For engagement (course), factor loadings ranged from 0.71-0.87, with 65.2% of the variance explained by a single factor. For engagement (lesson), factor loadings ranged from 0.73-0.89, with 68.3% of the variance explained by a single factor. For discomfort (course), factor loadings ranged from 0.83-0.95, with 82.5% of the variance explained by a single factor. For discomfort (lesson), factor loadings ranged from 0.89-0.95, with 84.5% of the variance explained by a single factor.

Evidence of validity based on relationships with other variables is found by examining correlations between measures from the different instruments (Supplemental Figure 1). The TTCI pre and post scores are highly and positively correlated with one another. All other measures also show a positive and significant correlation between overall measures regarding the course and overall measures regarding the lesson. Correlations between measures from different instruments show patterns that would be expected based on theory. For example, Engagement and Relevance measures are positively correlated, but both measures are negatively associated with discomfort. These associations are expected based on prior research on the importance of relevance to student motivation and engagement (Keller 1987, Frymier & Shulman 1995, Martin & Dowson 2009). Acceptance of human evolution is negatively associated with student discomfort, but positively associated with performance on the TTCI, which would also be predicted based on findings that low acceptance of evolution leads to feelings of alienation and discomfort in evolution courses (Hermann 2012, Barnes et al 2017).

Further evidence for validity of these measures is found in their original development, which in each case was performed with undergraduate student participants. The development of these instruments included tests of validity based on internal structure and based on relations to other variables was similarly found in the original generation of the relevance, engagement, discomfort, and evolution acceptance measures (Frymier & Shulman 1995, Richmond 1990, Barnes et al 2020, Nadelson & Southerland 2012).

Supplementary Table 1: Frequency of missing data patterns.

| **N**  (673) | **Course measures** | **Lesson measures** | **I-SEA** | **GPA** | **Mean GPA** |
| --- | --- | --- | --- | --- | --- |
| 275 (40.9) | Complete | Complete | Complete | Complete | 3.33 |
| 207 (30.8) | Complete | Missing | Complete | Complete | 3.04 |
| 74 (11) | Missing | Missing | Missing | Complete | 2.64 |
| 36 (5.3) | Missing | Complete | Complete | Complete | 3.14 |
| 27 (4) | Missing | Complete | Missing | Complete | 2.88 |
| 16 (2.4) | Complete | Missing | Missing | Complete | 2.50 |
| 9 (1.3) | Some missing | Complete | Complete | Complete | 3.11 |
| 8 (1.2) | Some missing | Missing | Complete | Complete | 2.38 |
| 6 (0.9) | Complete | Complete | Missing | Complete | 2.78 |
| 6 (0.9) | Missing | Some missing | Complete | Complete | 3.67 |
| 4 (0.6) | Missing | Missing | Complete | Complete | 3.08 |
| 2 (0.3) | Complete | Some missing | Complete | Complete | 3.50 |
| 2 (0.3) | Some missing | Missing | Missing | Complete | 2.67 |
| 1 (0.1) | Missing | Some missing | Missing | Complete | 3.67 |
|  | | | | | |

Supplementary Table 2: Main effects model results for student post-test TTCI scores.

|  | **TTCI Post-Score** | | | |
| --- | --- | --- | --- | --- |
|  | ***Complete case data*** | | ***Multiple imputation*** | |
|  | **Est**  **(std)** | **p-value** | **Est**  **(std)** | **p-value** |
| Intercept | **1.96 (1.44 – 2.48)** | **<0.001** | **1.88 (1.643 - 2.116)** | **<0.001** |
| TTCI Pre-Score | **0.59 (0.49 – 0.70)** | **<0.001** | **0.607 (0.559 - 0.655)** | **<0.001** |
| Year 2 (Reference: Year 1) | -0.41 (-0.82 – 0.01) | 0.057 | -0.238 (-0.409 - -0.067) | 0.164 |
| I-SEA (Centered) | 0.01 (-0.03 – 0.05) | 0.610 | 0.01 (-0.008 - 0.028) | 0.571 |
| Treatment – Human  (Reference: Animal) | 0.34 (-0.08 – 0.75) | 0.115 | 0.284 (0.088 - 0.480) | 0.150 |
| Observations | 282 | | 673 | |
| R^2^ / adjusted R^2^ | 0.342 / 0.332 | |  | |

Supplementary Table 3: Main effects model results for student reported engagement and content relevance during the one-day activity.

|  | **Engagement with lesson content** | | | | **Perceived relevance of lesson content** | | | |
| --- | --- | --- | --- | --- | --- | --- | --- | --- |
|  | ***Complete case data*** | | ***Multiple imputation*** | | ***Complete case data*** | | ***Multiple imputation*** | |
|  | **Est**  **(std)** | **p-value** | **Est**  **(std)** | **p-value** | **Est**  **(std)** | **p-value** | **Est**  **(std)** | **p-value** |
| Intercept | **4.85 (3.48 – 6.22)** | **<0.001** | **5.018 (4.325 - 5.711)** | **<0.001** | **8.07 (6.44 – 9.70)** | **<0.001** | **8.478 (7.711 - 9.244)** | **<0.001** |
| Engagement with course content | **0.59 (0.49 – 0.68)** | **<0.001** | **0.567 (0.518 - 0.616)** | **<0.001** | **-** | **-** | **-** | **-** |
| Perceived relevance of course content | **-** | **-** | **-** | **-** | **0.49 (0.38 – 0.59)** | **<0.001** | **0.448 (0.396 - 0.499)** | **<0.001** |
| Year 2 (Reference: Year 1) | -0.50 (-1.36 – 0.36) | 0.255 | -0.328 (-0.713 - 0.057) | 0.395 | -0.45 (-1.40 – 0.49) | 0.346 | -0.077 (-0.465 - 0.311) | 0.843 |
| I-SEA (Centered) | -0.02 (-0.10 – 0.06) | 0.553 | -0.001 (-0.038 - 0.037) | 0.989 | -0.01 (-0.10 – 0.08) | 0.891 | -0.014 (-0.052 - 0.024) | 0.712 |
| Treatment – Human  (Reference: Animal) | -0.34 (-1.17 – 0.50) | 0.426 | -0.43 (-0.830 - -0.029) | 0.285 | -0.19 (-1.14 – 0.75) | 0.688 | -0.467 (-0.906 - -0.028) | 0.289 |
| Observations | 281 | | 673 | | 285 | | 673 | |
| R^2^ / adjusted R^2^ | 0.365 / 0.356 | |  | | 0.237 / 0.226 | |  | |

Supplementary Table 4: Full model results for student reported discomfort experienced during the one-day activity.

|  | **Discomfort with lesson content** | | | |
| --- | --- | --- | --- | --- |
|  | ***CC*** | | ***MI*** | |
|  | ***Β***  **(Std. error)** | **p-value** | ***Β***  **(Std. error)** | **p-value** |
| Intercept | **0.16 (0.09 – 0.29)** | **<0.001** | **0.17 (-1.13 - 1.47)** | **<0.001** |
| Discomfort with course content | **1.88 (1.60 – 2.22)** | **<0.001** | **1.903 (0.82 - 2.98)** | **<0.001** |
| Year  (Reference: Year 1) | 1.32 (0.71 - 2.46) | 0.379 | 1.269 (-0.01 - 2.55) | 0.338 |
| I-SEA  (Centered) | 0.93 (0.85 – 1.01) | 0.073 | 0.938 (-0.1 - 1.97) | 0.076 |
| Treatment (Reference: Animal) | 0.88 (0.47 – 1.62) | 0.676 | 0.841 (-0.47 - 2.15) | 0.524 |
| Treatment X  I-SEA | 0.99 (0.87 – 1.13) | 0.914 | 1.001 (-0.05 - 2.05) | 0.979 |
| Observations | 285 | | 673 | |
| R^2^ / adjusted R^2^ | 0.323 / 0.442 | |  | |
| Full model results predicting student discomfort using complete-case data (CC) and multiple imputation (MI). The logistic regression models the likelihood students reported a discomfort score above zero. Odds ratios are reported for the discomfort model. Year and treatment are binary variables with reference values in parentheses. MI results are pooled following (Rubin 2004). Coefficients significant at p ≤ 0.05 are bolded. | | | | |

# Diagnostic checks for imputation

The following figures display the imputed values against known values for each imputation. The first facet (0) displays the known values, with the probability of data missing on the X axis and the variable measure on the Y axis. These figures show that imputed values fall within the range of real values given the propensity that a data point is missing.

**
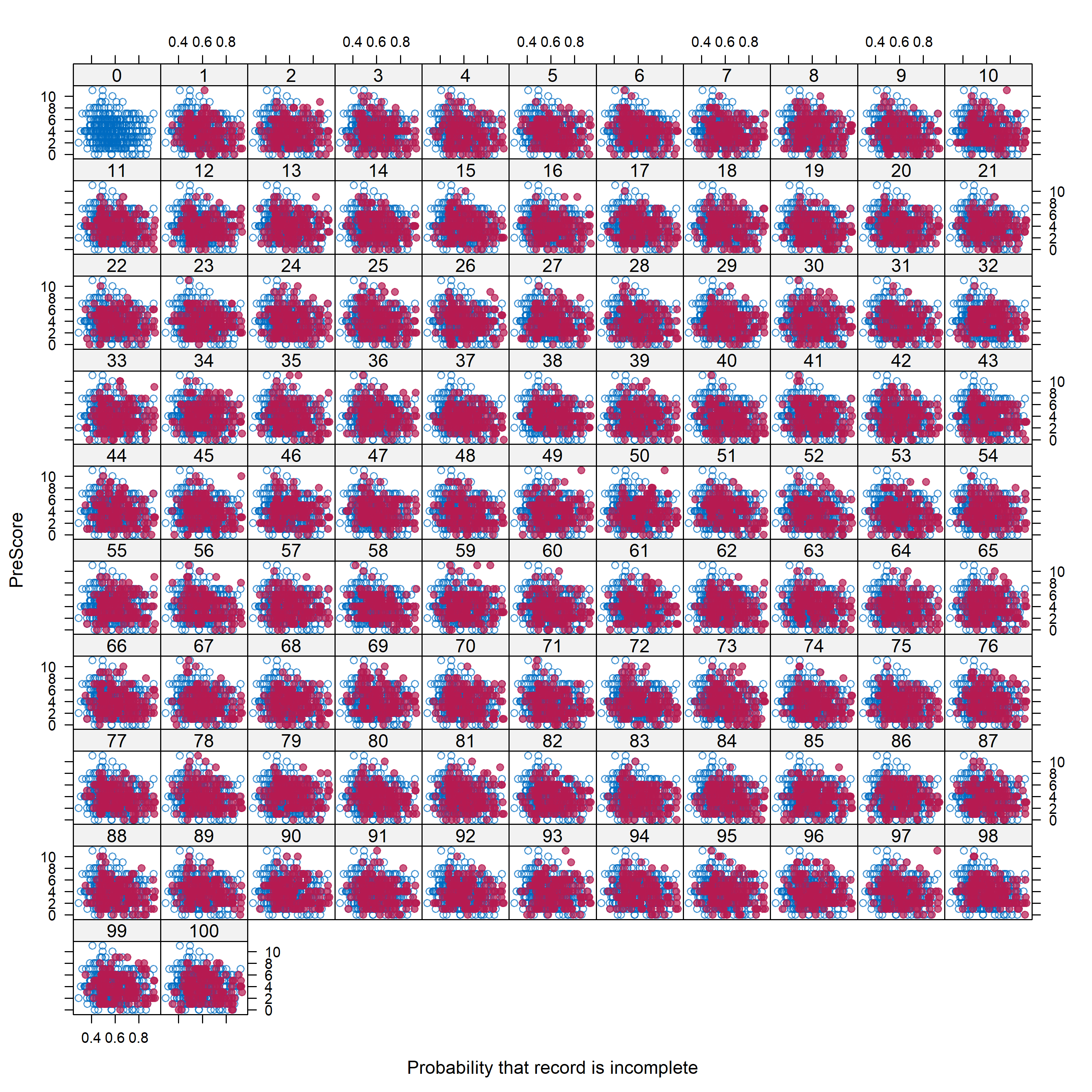
**

Supplementary Figure 3: Observed TTCI Pre- scores (blue) and imputed values for the 100 datasets (red).

**
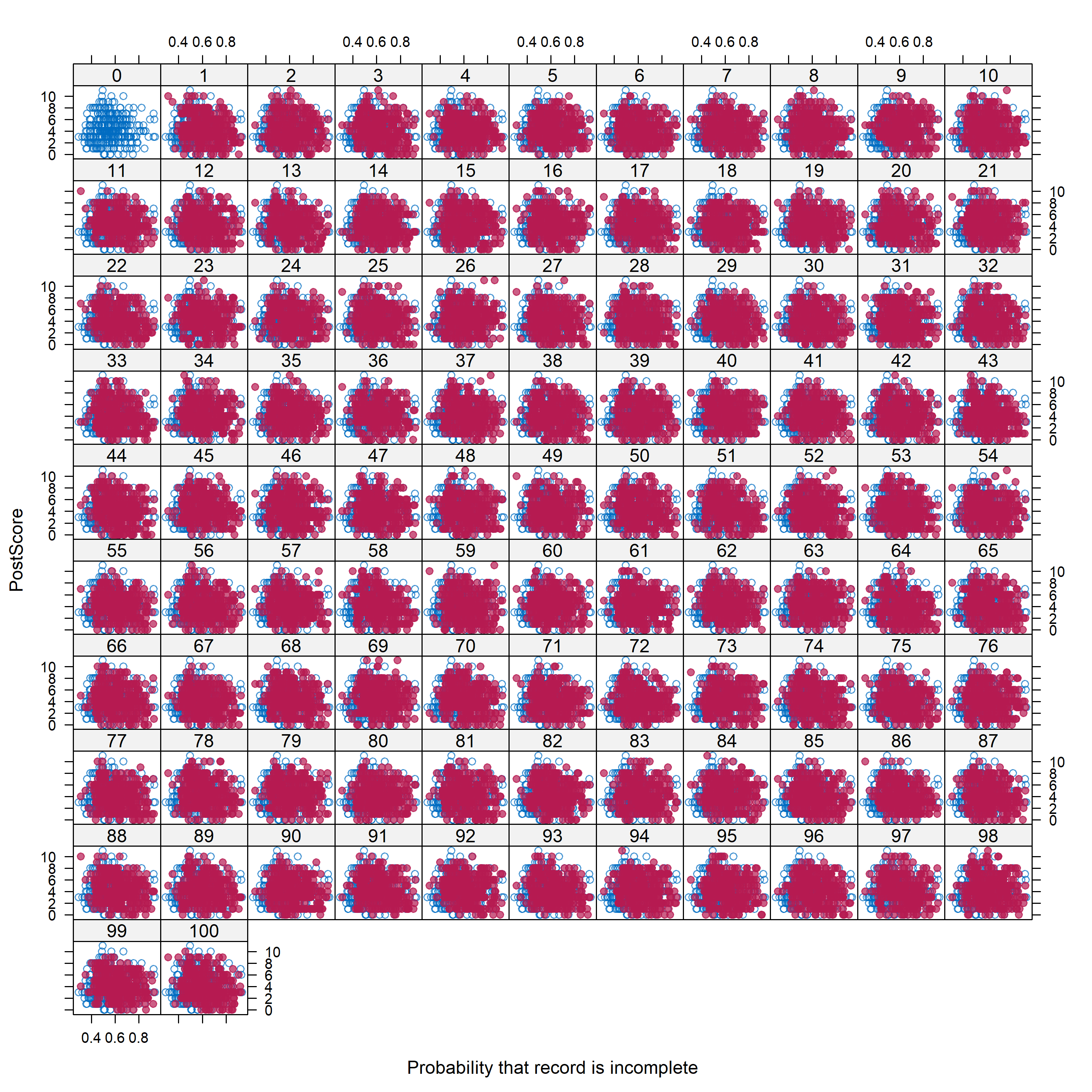
**

Supplementary Figure 4: Observed TTCI Post- scores (blue) and imputed values for the 100 datasets (red).

**
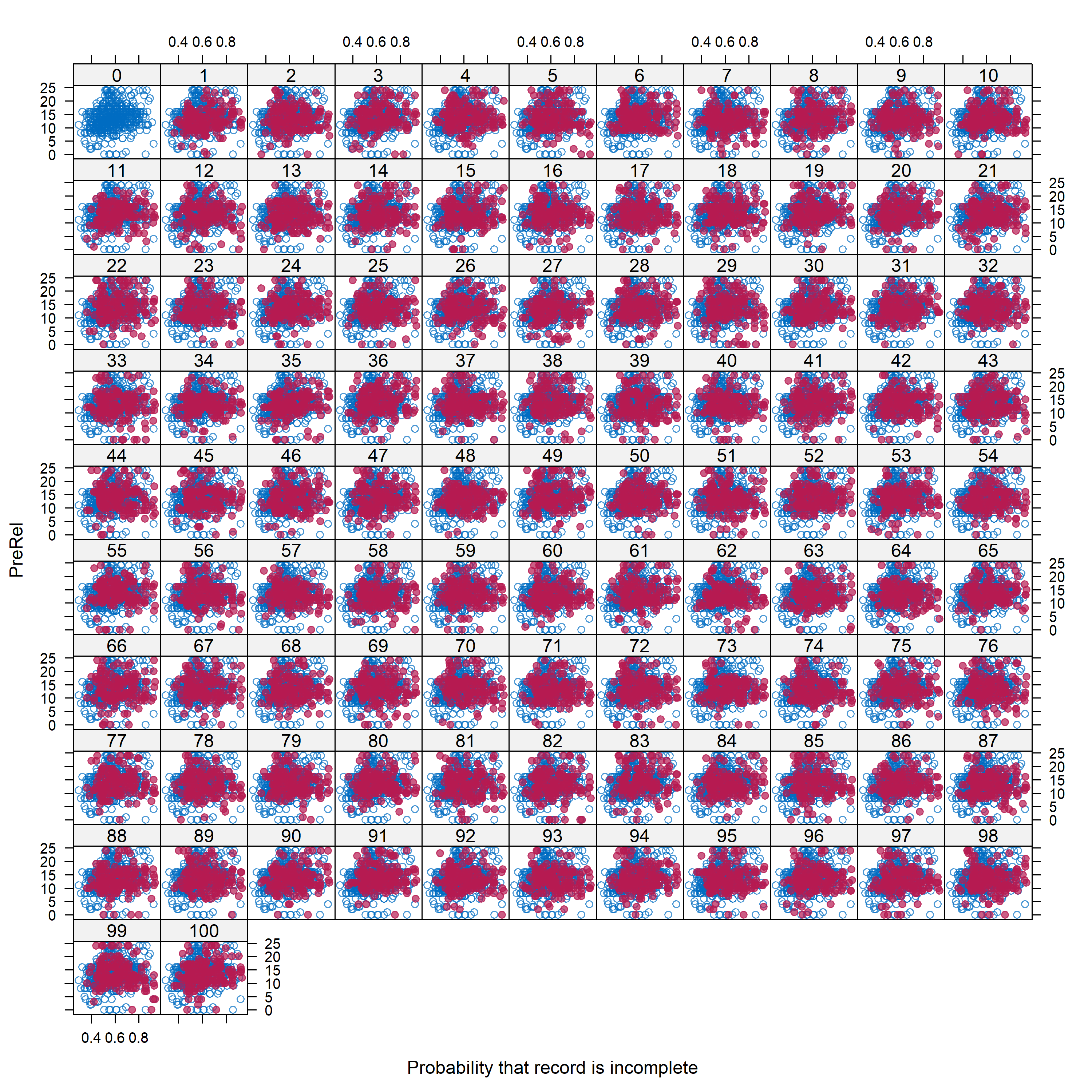
**

Supplementary Figure 5: Observed Relevance (course) scores (blue) and imputed values for the 100 datasets (red).

**
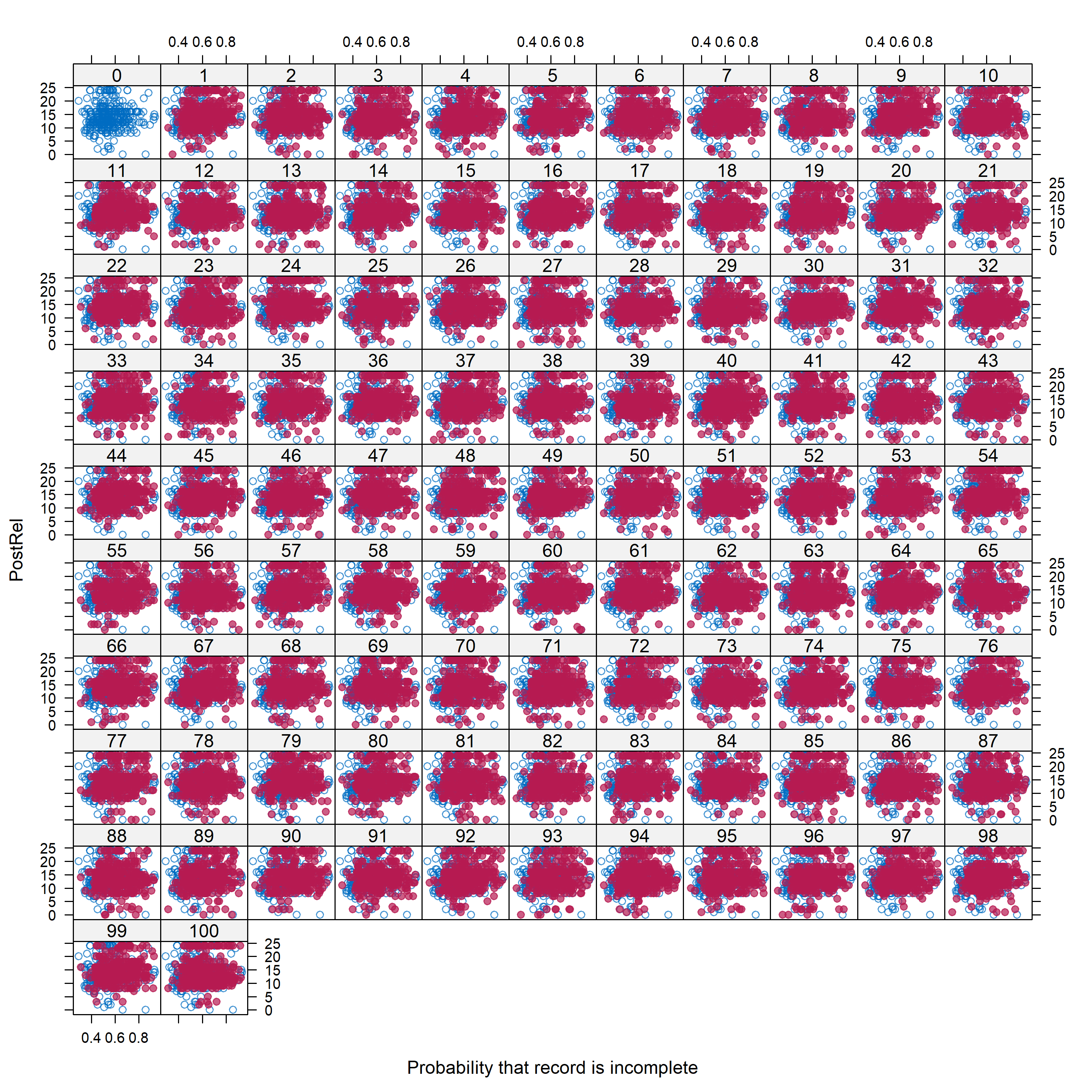
**

Supplementary Figure 6: Observed Relevance (lesson) scores (blue) and imputed values for the 100 datasets (red).

**
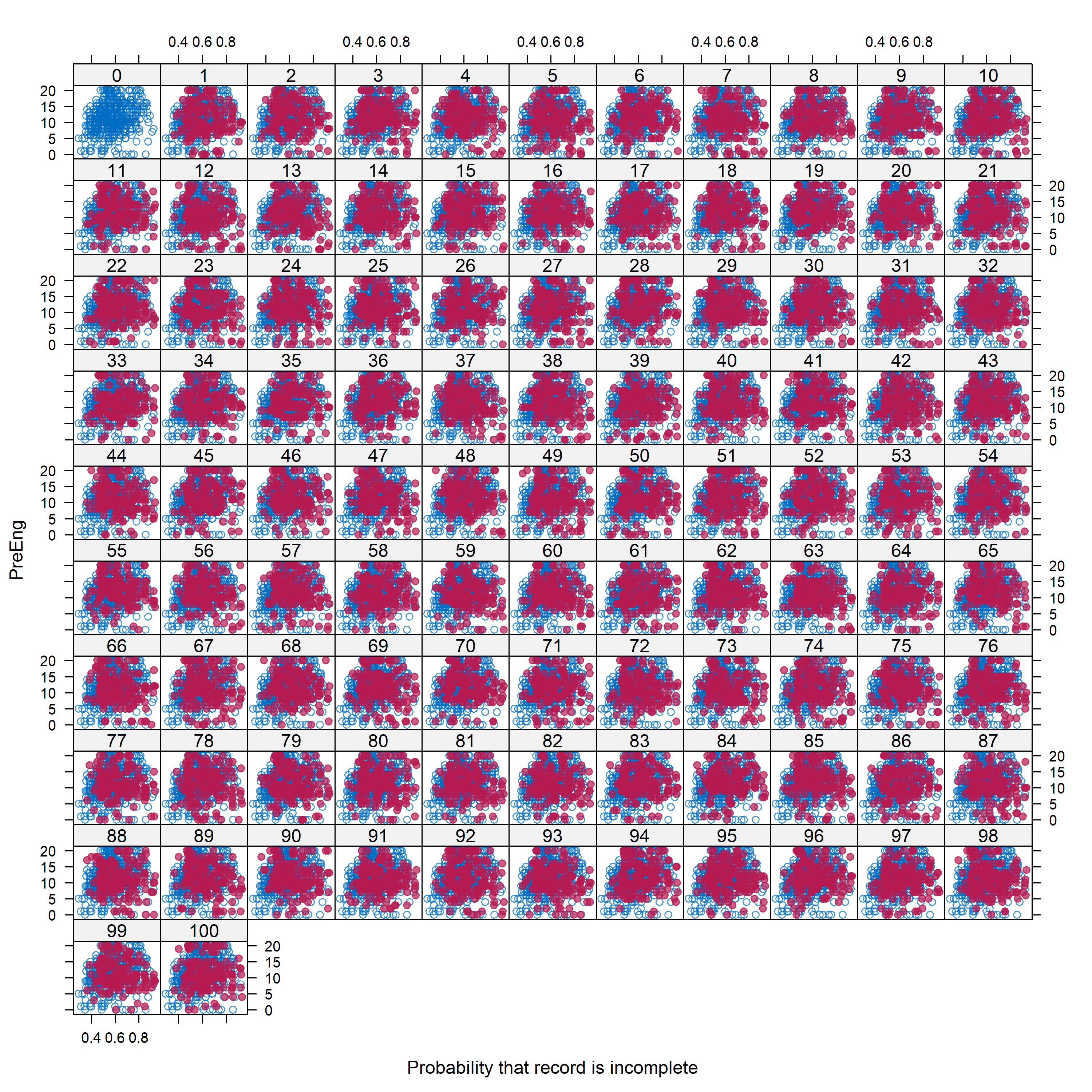
**

Supplementary Figure 7: Observed Engagement (course) scores (blue) and imputed values for the 100 datasets (red).

**
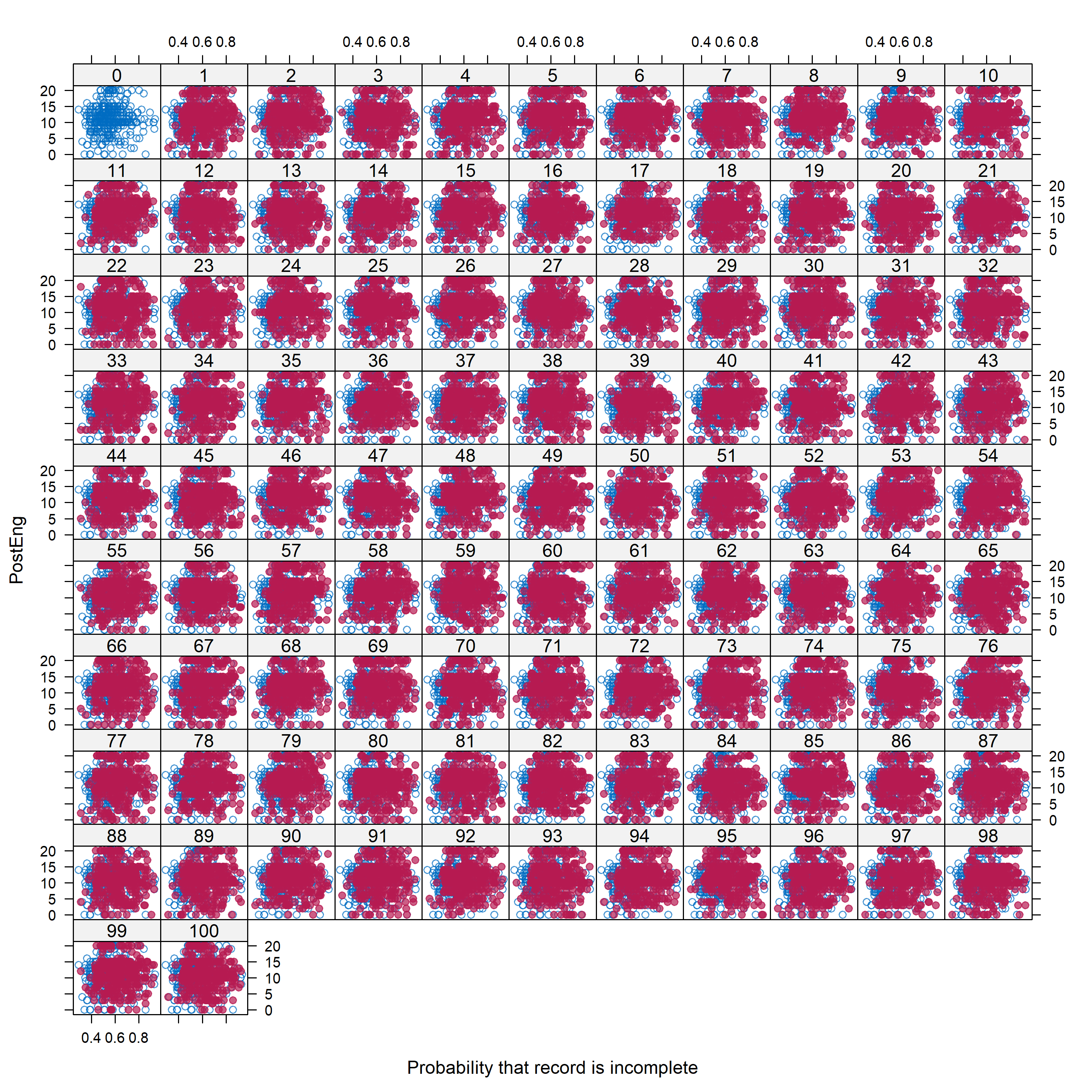
**

Supplementary Figure 8: Observed Engagement (lesson) scores (blue) and imputed values for the 100 datasets (red).

**
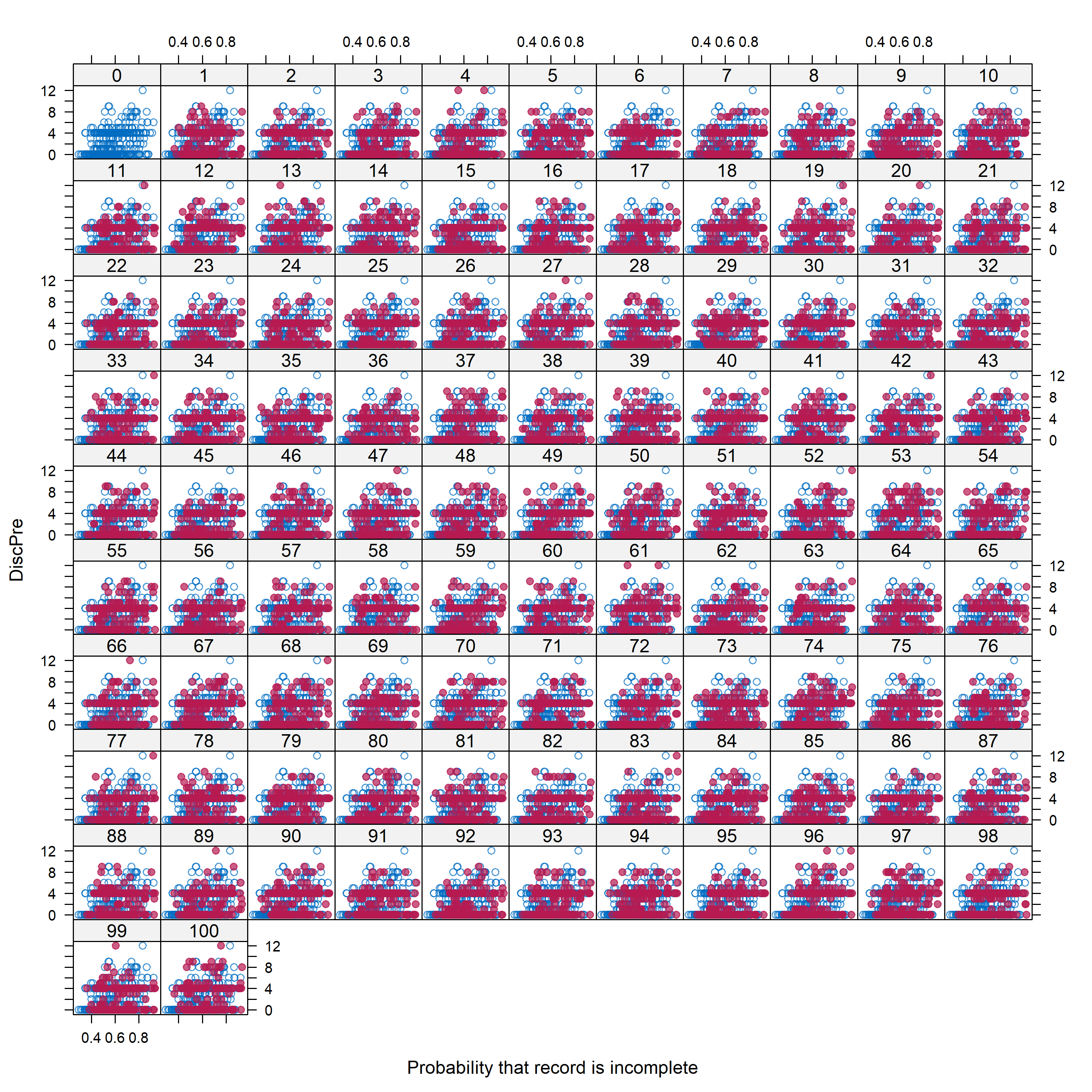
**

Supplementary Figure 9: Observed Discomfort (course) scores (blue) and imputed values for the 100 datasets (red).

**
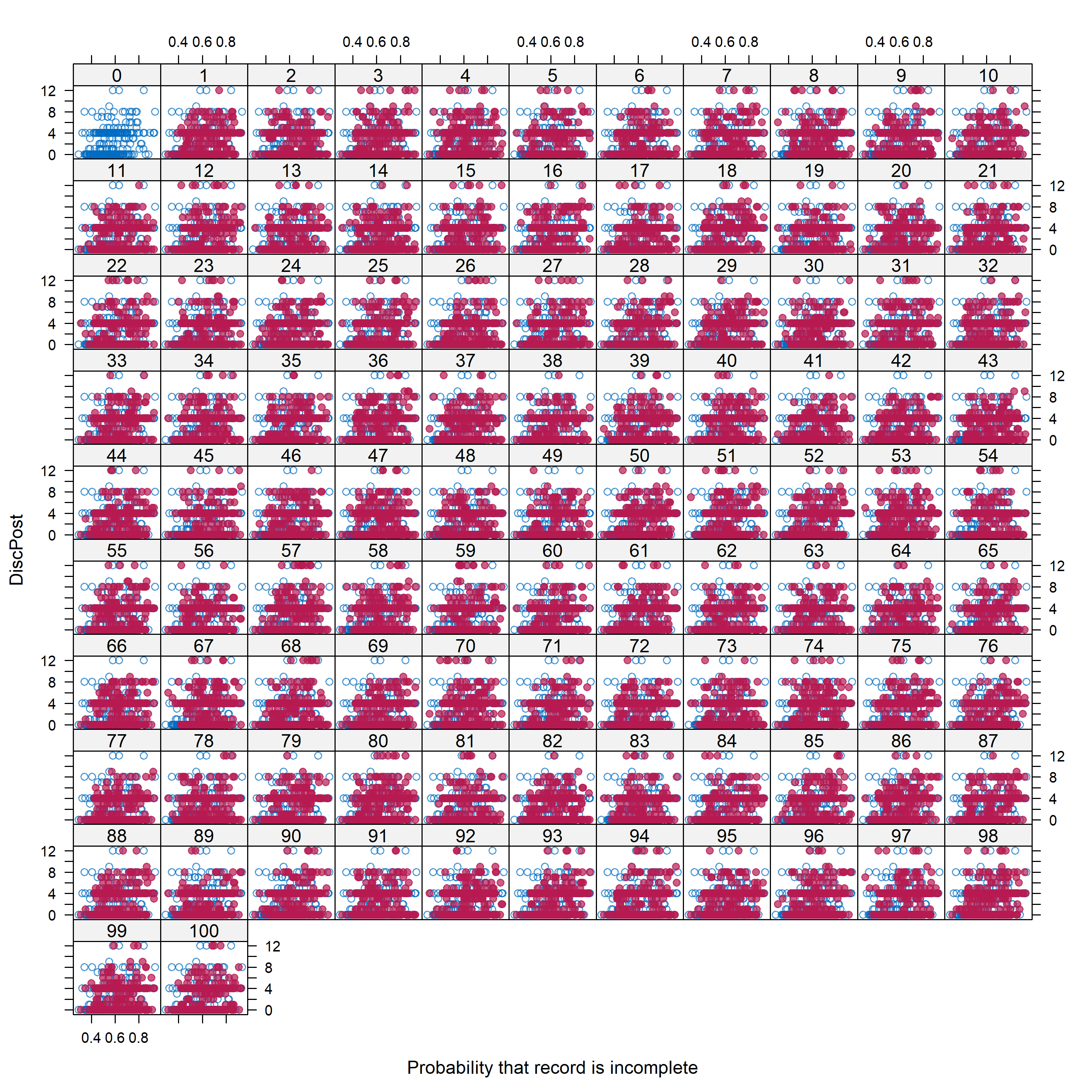
**

Supplementary Figure 10: Observed Discomfort (lesson) scores (blue) and imputed values for the 100 datasets (red).

The following figures show the density of the imputed values for each of the 100 imputed datasets. Each plot contains a blue line indicating the distribution from the complete dataset, though this line is hard to see behind the lines indicating the imputation distributions. Generally, imputations follow the same distribution as complete case data.


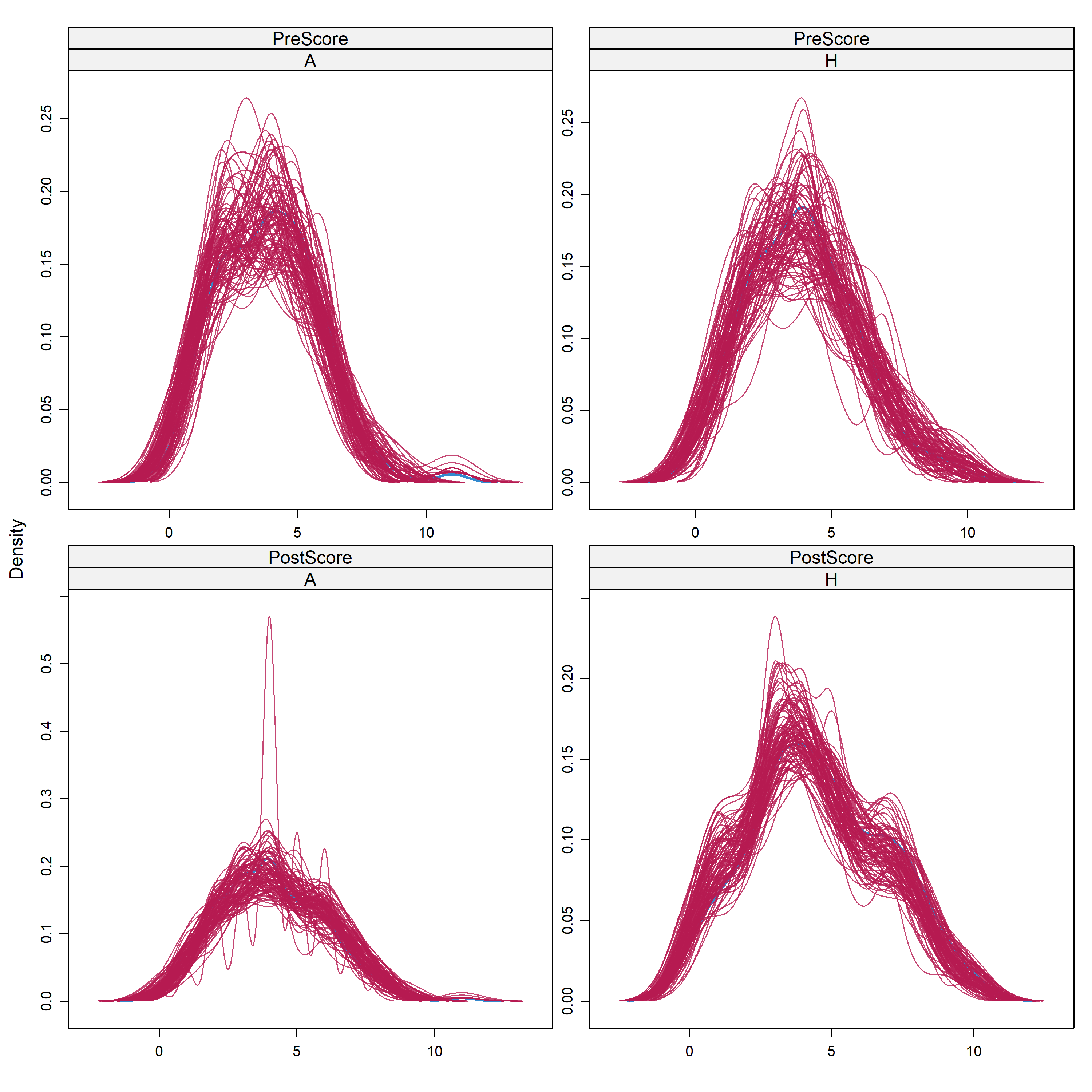


Supplementary Figure 11: Distribution of imputed TTCI scores for 100 datasets (red) compared to observed scores (blue).

**
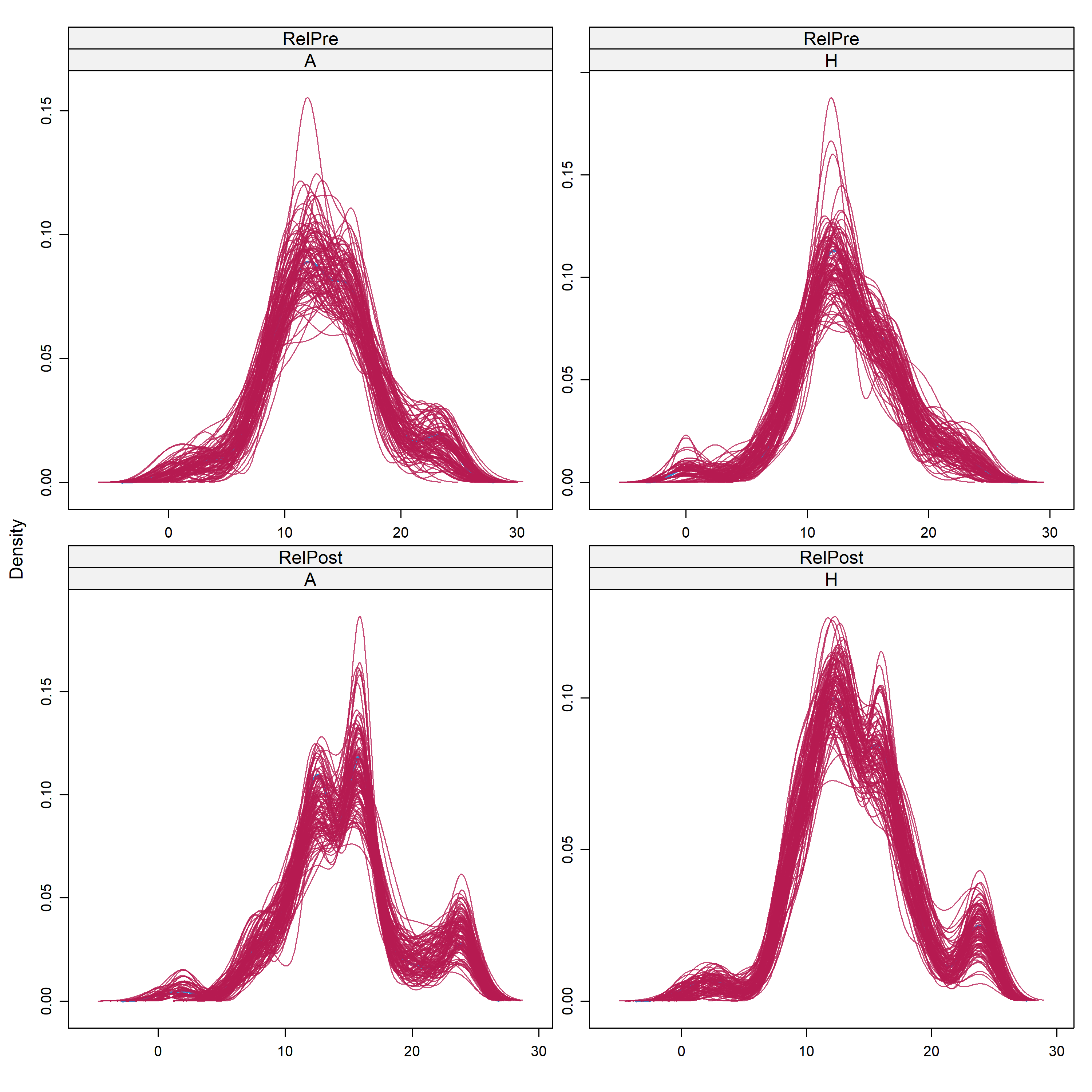
**

Supplementary Figure 12: Distribution of imputed Relevance scores for 100 datasets (red) compared to observed scores (blue). RelPre refers to perceived relevance of the course content and RelPost refers to perceived relevance of the lesson content.

**
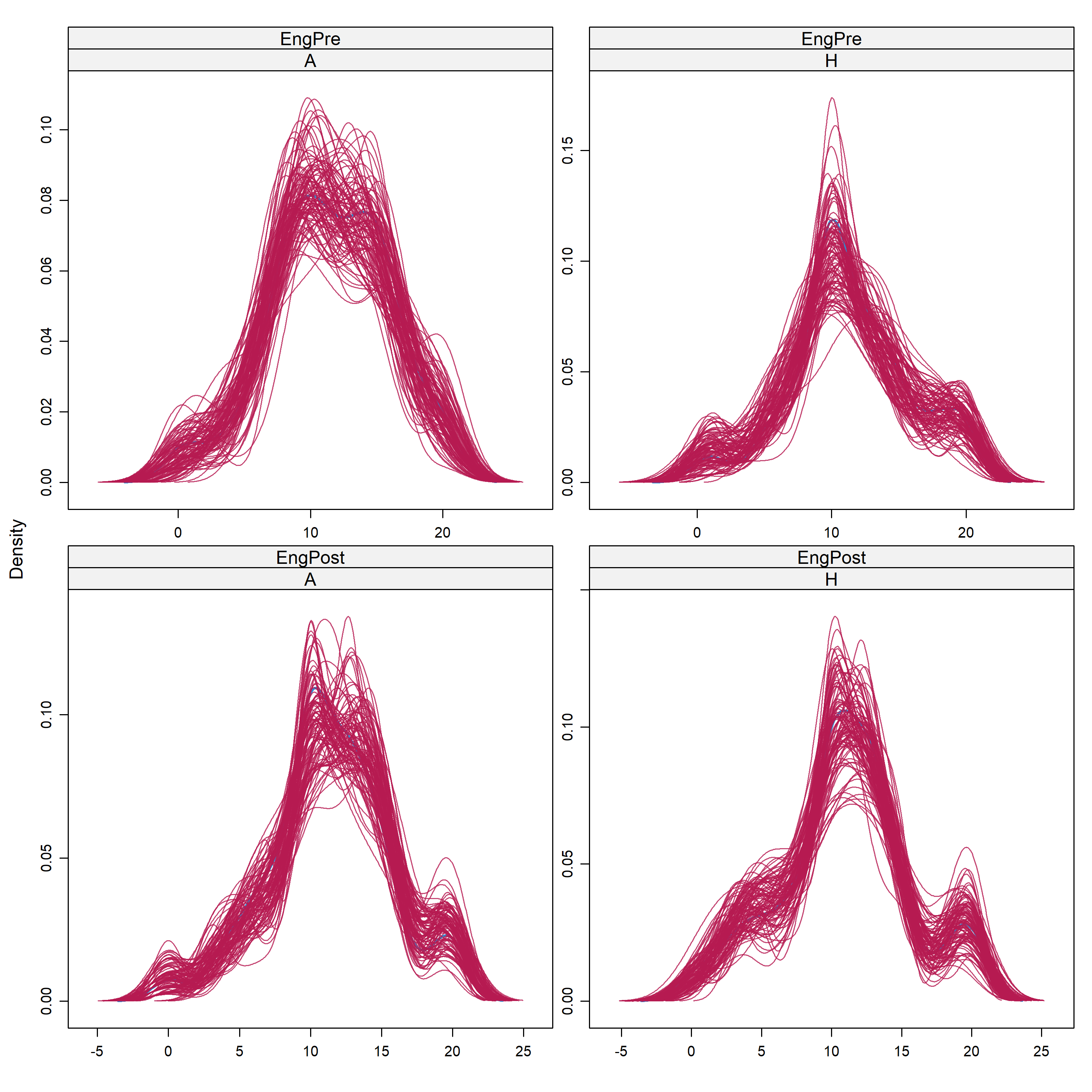
**

Supplementary Figure 13: Distribution of imputed Engagement scores for 100 datasets (red) compared to observed scores (blue). EngPre refers to engagement with the course content and EngPost refers to Engagement with the lesson content.

**
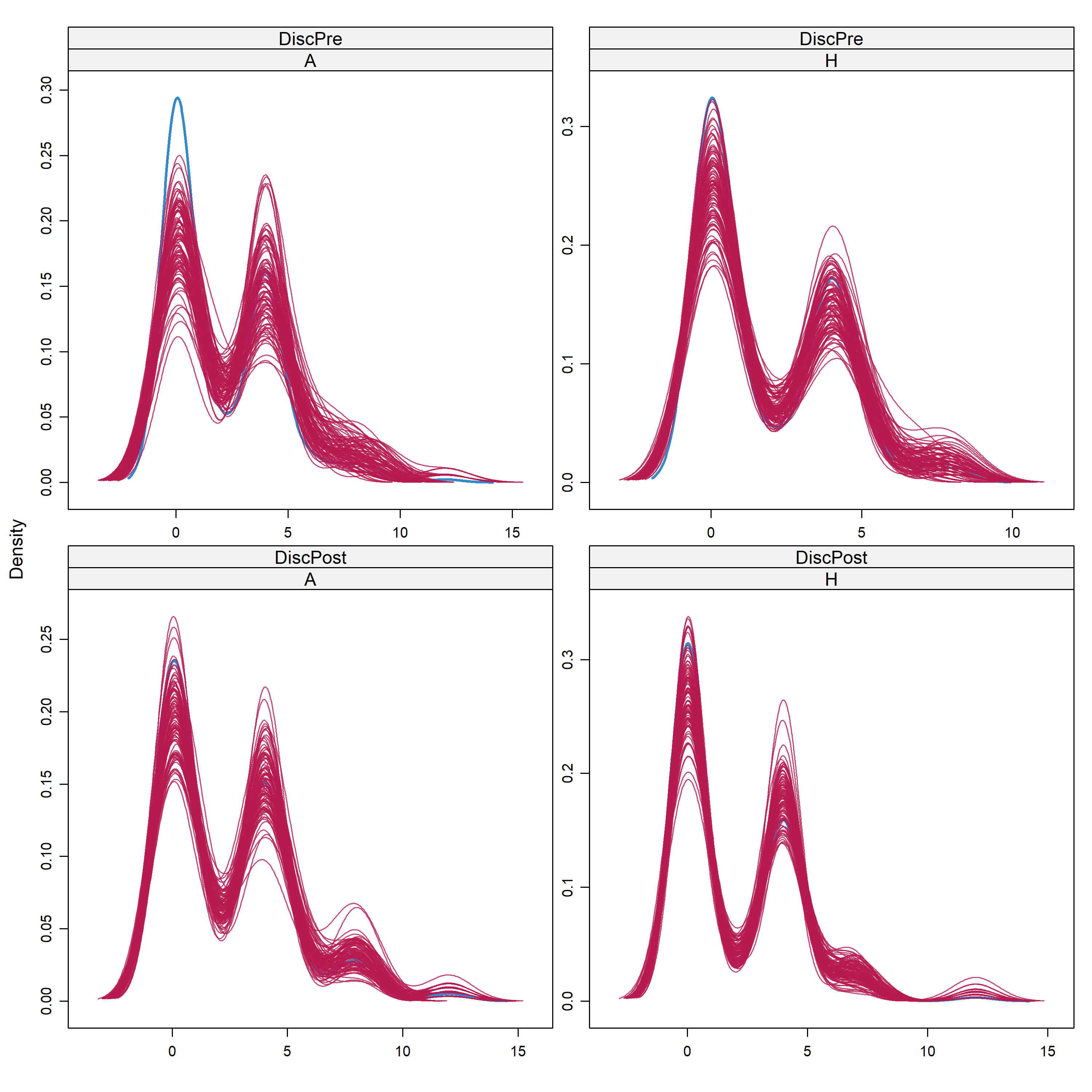
**

Supplementary Figure 14: Distribution of imputed Discomfort scores for 100 datasets (red) compared to observed scores (blue). DiscPre refers to discomfort with the course content and DiscPost refers to discomfort with the lesson content.
